# Unlocking Electrochemical-Driven Surface Oxygen Vacancies-Regulated Cathode–Electrolyte Interphase for Stabilizing Li-Ion Cells

**DOI:** 10.1007/s40820-026-02170-3

**Published:** 2026-05-09

**Authors:** Chenxi Yan, Xing Liu, Xuanlong He, Junjie Han, Longjun He, Na Tian, Yanyi Wang, Qiang Li, Ning Zhao, Lipeng Zhang, Peixin Zhang, Dingtao Ma

**Affiliations:** 1 College of Chemistry and Environmental Engineering, Shenzhen University, Shenzhen, 518060 People’s Republic of China; 2 School of Materials and New Energy, South China Normal University, Shanwei, 516600 People’s Republic of China; 3 College of Materials and Environmental Engineering, Shenzhen Polytechnic University, Shenzhen, 518055 People’s Republic of China

**Keywords:** Cathode material, Oxygen vacancies, Cathode–electrolyte interphase, LiCoO_2_, Li_2_C_4_O_4_

## Abstract

A stable cathode–electrolyte interphase (CEI) significantly enhances the durability of lithium-ion batteries; however, the intricate chemistry underlying its formation makes predesign exceedingly challenging. This work demonstrates that surface oxygen vacancy (OV) concentration dually regulates CEI thickness and composition. We develop an in situ strategy where Li_2_C_4_O_4_ incorporation into LiCoO_2_ (LCO) spontaneously decomposes during cycling, generating surface OVs. Combined experimental and theoretical calculations reveal these OVs enhance interfacial electron/Li⁺ migration rates while stabilizing the CEI. Notably, through ^18^O isotope labeling with time-of-flight secondary ion mass spectrometry, we innovatively provide direct experimental evidence that surface lattice oxygen serves as the predominant oxygen source for CEI oxygen-containing decomposition products, establishing the mechanism for OV-mediated CEI modulation. Based on this theory, the linear correlation and causal relationship among Li_2_C_2_O_4_ content, surface OV concentration, and LiF/Li_x_PO_y_F_z_ ratio in CEI are revealed. This strategy endows the OV-rich LCO cathode with 71.1% capacity retention after 600 cycles at 1 C within 3–4.4 V (23.9% for bare LCO) and achieves universal validation at 4.5 V and in LiNi_0.8_Co_0.1_Mn_0.1_O_2_ (NCM811). This study elucidates the critical function of surface OVs in prolonging cycle life and establishes a new design principle for tailored cathode interfaces and CEI chemistry.

**Supplementary Information:**

The online version contains supplementary material available at 10.1007/s40820-026-02170-3.

## Highlights

- In situ electrochemically induced surface oxygen vacancies (OVs) by Li_2_C_4_O_4_ decomposition enable dual regulation of cathode electrolyte interphase (CEI) thickness and composition, a novel strategy for tailored CEI engineering in Li ion batteries.
- For the first time,^18^O isotope labeling combined with TOF SIMS directly verifies that lattice oxygen of LiCoO_2_ (LCO) serves as the predominant oxygen source for Li_x_PO_y_F_z_ in CEI, clarifying the long debated LiPF_6_ decomposition mechanism at the cathode inte rface.
- The OV mediated strategy endows LCO with 71.1% capacity retention after 600 cycles at 1 C (3–4.4 V) (vs. 23.9% for bare LCO) and achieves universal applicability in 4.5 V high voltage systems and NCM811 cathodes, establishing a new design principle for stable high performance cathodes.

**Supplementary Information:**

The online version contains supplementary material available at 10.1007/s40820-026-02170-3.

## Graphical abstract

**Figure:**
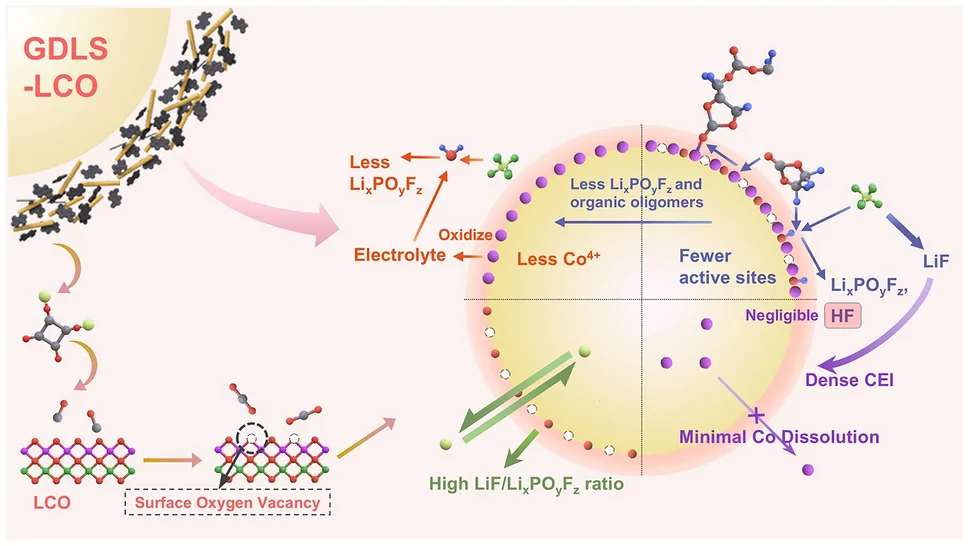

## Introduction

The growing societal demand for clean and renewable energy sources has led to the increased adoption of lithium-ion batteries (LIBs), prized for their high energy density and long cycle life [1–3]. The optimization of the cathode is of paramount importance for enhancing the performance of LIBs [4]. Among cathode materials, lithium-containing transition metal oxides, such as LiCoO_2_ (LCO), remain widely used yet offer significant room for improvement in key areas like energy density and cycling stability. During battery cycling, the accumulation of reaction products from electrolyte decomposition leads to the formation of a cathode–electrolyte interphase (CEI) layer, which evolves with successive cycles. Investigations have revealed that the interface of the cathode material and its accompanying CEI layer critically influences the battery's cycle performance [5–9]. A thin, uniform, and robust CEI layer is generally acknowledged to be effective in mitigating cathode degradation. It is hoped that the CEI layer can be "designable" by controlling interfacial reactions, but the complexity of interfacial chemical reactions makes this difficult to achieve. Current optimization strategies are largely empirical, with well-established design principles remaining scarce. Consequently, identifying effective methods to engineer the CEI layer is of paramount importance.

Oxygen vacancies (OVs) were initially viewed as detrimental, indicative of lattice oxygen loss, whereas numerous recent studies have revealed that an appropriate concentration of OVs can confer multifaceted benefits to battery performance, including the reduction in oxidized transition metal valence states, protection of the cathode against irreversible phase transitions, and enhancement of Li^+^ ionic conductivity [10–12]. Cai et al. further proposed a surface OV-rich layer acting as a "buffer" for the bulk lattice oxygen, demonstrating its efficacy in suppressing the loss of internal lattice oxygen [13]. The role of surface OVs extends beyond this; they also significantly influence the adjacent CEI layer. Wang et al. found that surface OVs effectively suppress the formation of surface O–O dimers, thereby mitigating parasitic side reactions and promoting the formation of a dense CEI [14]. We posit that surface OVs represent a key to regulating the CEI layer. Nevertheless, research on how surface OVs influence the CEI remains limited, and the underlying theory is neither clear nor unified. Therefore, elucidating the fundamental mechanisms is critically important.

The CEI layer possesses a complex composition, broadly categorized into organic and inorganic components. This work focuses on LiF and oxygen-containing decomposition products like Li_x_PO_y_F_z_, derived from LiPF_6_ hydrolysis. These species constitute a significant fraction of the CEI and represent distinct decomposition pathways of LiPF_6_. A high LiF content is widely believed to facilitate a dense, stable, corrosion-resistant, and ionically conductive CEI, which is crucial for protecting the electrode and enhancing interfacial kinetics [15–17]. In contrast, oxygen-containing species like Li_x_PO_y_F_z_ exhibit inferior robustness and conductivity compared to LiF and are often accompanied by the generation of detrimental organic oligomers and HF [18, 19]. Thus, a high LiF/Li_x_PO_y_F_z_ ratio is desirable for an optimal CEI, necessitating the suppression of Li_x_PO_y_F_z_ formation. Since LiPF_6_ itself contains no oxygen, the generation of Li_x_PO_y_F_z_ requires an external oxygen source. Controlling this source is key to modulating its formation. Early studies attributed the oxygen atoms in Li_x_PO_y_F_z_ primarily to H_2_O from the decomposition of electrolyte [20]. However, recent research suggests that surface lattice oxygen, possessing higher reactivity, is a more predominant oxygen source for these species. Spotte-Smith et al. supported this through computational studies, showing LiPF_6_'s preferential reaction with highly active oxygen sites, such as lattice oxygen atoms at the transition metal oxide interface, which act as Lewis bases [21]. Furthermore, numerous studies suggest that electrolyte molecules (e.g., ethylene carbonate, EC) adsorb onto the cathode surface, where their hydrogen atoms can interact with surface lattice oxygen to form hydroxyl groups. These highly reactive hydroxyls then react with PF_6_^−^ to form Li_x_PO_y_F_z_ and HF [18, 19, 22–24]. Nonetheless, direct experimental evidence confirming this pathway has been lacking. To address this, we employed ^18^O isotope labeling combined with time-of-flight secondary ion mass spectrometry (TOF–SIMS) to trace the fate of lattice oxygen. This approach definitively identifies surface lattice oxygen as a significant oxygen source for Li_x_PO_y_F_z_ species in the CEI. We also demonstrate that in situ-generated surface OVs on LCO, induced by the spontaneous decomposition of Li_2_C_2_O_4_ during cycling, can effectively regulate the formation of Li_x_PO_y_F_z_.

In this work, we confirm that a high concentration of surface OVs significantly enhances interfacial kinetics and optimizes both the thickness and composition of the CEI. Guided by this principle, the designed LCO delivers a remarkable capacity retention of 71.1% after 600 cycles at 1 C, substantially outperforming pure LCO (23.9%). It maintains considerable capacity and cycling stability. The regulation mechanism of surface OVs on the CEI not only enables long-term cyclability of LCO-based batteries but also provides a crucial design principle for the targeted engineering of the hitherto elusive CEI.

## Experimental Section

### Material

H_2_C_4_O_4_ (≥ 98%, Aladdin), Li_2_CO_3_ (99.5%, Macklin), graphite (≥ 98%, Aladdin), H_2_^18^O (≥ 97 atom% ^18^O, Aladdin), Co_3_O_4_ powder (≥ 99.9%, Aladdin), lithium-ion battery electrolyte (1 M LiPF_6_ in DEC:EC:EMC = 1:1:1 by volume with 5% FEC), lithium metal foils were purchased from Dongguan CANRD New Energy Technology Co., Ltd. All the above materials were used directly without further purification.

### Material Synthesis

#### Synthesis of DLS and GDLS

H_2_C_4_O_4_ and Li_2_CO_3_ were employed as starting materials at a molar ratio of 1.03:1, with H_2_C_4_O_4_ in slight excess. Initially, H_2_C_4_O_4_ was added to and dissolved in deionized water at 60 °C under stirring until a colorless, transparent solution was obtained. After 10 min, the pre-ground Li_2_CO_3_ powder was introduced into the solution. The mixture was maintained at 60 °C with continuous stirring for 1 h. Subsequently, the solution was transferred to an oven and dried at 80 °C. The resulting solid was washed with anhydrous ethanol and then dried again at 80 °C to yield the final DLS powder. The GDLS composite was prepared by mechanically mixing the as-synthesized DLS with flake graphite. The precursors (DLS and graphite at a mass ratio of 3:2) were first individually ground and then subjected to a high-speed ball-milling process for 10 min to achieve a homogeneous mixture (GDLS).

#### Synthesis of LiCo^18^O_x_O_2-x_

A mixture of H₂^18^O and deionized water (volume ratio 1:4) was first prepared. Lithium metal foils were added cautiously to this mixture (piece by piece to mitigate the vigorous reaction) until the reaction was completed. Excess lithium metal was then removed. CO_2_ gas was bubbled through the resulting solution until a thick white suspension formed and no further thickening was observed. The suspension was dried at 80 °C to obtain a solid powder, which was subsequently washed with anhydrous ethanol and dried again at 80 °C to yield Li_2_C^18^O_x_O_3-x_. This intermediate product was mixed with Co_3_O_4_ at a stoichiometric ratio of Li/Co = 1.03, followed by thorough grinding. The blended powder was heated in air at a ramp rate of 3 °C min^−1^ to 900 °C, held at this temperature for 15 h, and finally ground to obtain the target material LiCo^18^O_x_O_2-x_.

### Material Characterizations

The surface morphology and elemental distribution were characterized by field emission scanning electron microscopy (FE-SEM) (JEOL, JSM-7800F) equipped with an energy-dispersive X-ray spectroscopy (EDS) detector (Ametek, TEAMOctane Plus) operating at 15 kV. Transmission electron microscopy (TEM) was carried out on a JEM-2100 & X-Max80 instrument at an acceleration voltage of 200 kV. X-ray diffraction (XRD) patterns were collected on a PANalytical Empyrean diffractometer with Cu Kα radiation (λ = 1.54065 Å), using a generator setting of 45 kV and 40 mA. Fourier transform infrared (FTIR) spectra were acquired on a Thermo Fisher Nicolet iS50 spectrometer. In situ differential electrochemical mass spectrometry (DEMS) was performed using a GAS100-Li system (Shanghai Linglu Instrument Equipment Co., Ltd.). X-ray photoelectron spectroscopy (XPS) measurements were conducted on a Thermo Fisher Scientific K-Alpha instrument with Al Kα radiation, using a step size of 0.05 eV. High-resolution TEM (HRTEM) imaging and electron energy loss spectroscopy (EELS) line scans were obtained on an FEI Tecnai G2 F30 microscope. Electron paramagnetic resonance (EPR) spectroscopy was performed on a Bruker E500 spectrometer. Time-of-flight secondary ion mass spectrometry (TOF–SIMS) 3D reconstructions were generated using an ION TOF–SIMS 5 system.

### Electrochemical Performance Measurements

The cathode slurry was prepared by uniformly mixing active material, acetylene black, and PVDF at a mass ratio of 8:1:1 (for DLS||Li and GDLS||Li cells, the ratio was adjusted to 6.5:2.5:1) in N-methyl-2-pyrrolidone (NMP), followed by coating onto aluminum foil and vacuum-drying at 80 °C for 10 h. CR2032-type coin cells were used for all battery assembly and electrochemical tests. The material loading was controlled within 1.5–2.0 mg cm^−2^. For full cells, the anode was fabricated using graphite, acetylene black, and PVDF at a mass ratio of 8:1:1, coated onto copper foil with procedures identical to those of the cathode. The negative/positive (N/P) ratio was designed between 1.05 and 1.15. All cells were assembled in an argon-filled glove box with H₂O and O₂ levels below 0.01 ppm. During testing, the 1 C current density was defined as 425 mAh g^−1^ for DLS electrodes, and 170 mAh g^−1^ for LCO and GDLS-LCO electrodes. All cells underwent activation by three cycles at 0.1 C. Polypropylene (PP) membrane was used as the separator in all configurations. For full-cell assembly, graphite anodes require offline prelithiation before cycling. Specifically, the graphite electrode was first assembled into a half-cell and cycled three times at 0.1 C (1 C = 350 mAh g^−1^) after which it was extracted and used to construct the full cell. All charge–discharge tests, except those conducted at − 30 °C, were performed at room temperature (25 °C) using a LAND CT3001A battery test system. The galvanostatic intermittent titration technique (GITT) tests were performed using a 20-min titration step at 0.1 C and a 2-h relaxation step. Cyclic voltammetry (CV) and electrochemical impedance spectroscopy (EIS) measurements were carried out on a Solartron Analytical 1470E station (Ametek) with an amplitude of 10 mV at a frequency of 1000 kHz.

### Density Functional Theory Calculation

The density functional theory (DFT) calculations were carried out with the VASP code [25]. The Perdew–Burke–Ernzerhof (PBE) functional within generalized gradient approximation (GGA) was used to process the exchange–correlation [26], while the projector-augmented-wave pseudopotential (PAW) was applied with a kinetic energy cutoff of 500 eV [27], which was utilized to describe the expansion of the electronic eigenfunctions. The vacuum thickness was set to be 15 Å to minimize interlayer interactions. The Brillouin-zone integration was sampled by a Γ-centered 7 × 7 × 1 Monkhorst–Pack k-point. All atomic positions were fully relaxed until energy and force reached a tolerance of 1 × 10^–6^ eV and 0.01 eV Å^−1^, respectively. The dispersion-corrected DFT-D method was employed to consider the long-range interactions [28]. Employing the climbing image nudged elastic band method (CI-NEB), we computed the minimum energy pathway of the diffuse reaction along with its corresponding activation barrier.

The Gibbs free energy change (ΔG) was calculated by computational hydrogen electrode (CHE) model as follows:

1 $$\Delta {\mathrm{G}} = \Delta {\mathrm{E}} + \Delta {\mathrm{ZPE}}{-}{\mathrm{T}}\Delta {\mathrm{S}}$$

where ΔE is the reaction energy obtained by the total energy difference between the reactant and product molecules absorbed on the catalyst surface and ΔS is the change in entropy for each reaction, and ΔZPE is the zero-point energy correction to the Gibbs free energy.

## Result and Discussion

### Enhanced Cycling Performance via Li_2_C_4_O_4_ Incorporation

While dilithium squarate (Li_2_C_4_O_4_, DLS) is primarily used as a cathode prelithiation agent in lithium-ion batteries [29, 30], its ability to generate surface OVs has not been reported previously. The original aim of this study was to investigate the prelithiation effect of DLS on LiCoO_2_ (LCO). Owing to the inherently poor electronic conductivity of DLS, its direct incorporation into LCO cathode materials would likely impair battery performance. In line with the principle of simplicity for synthesizing cathode prelithiation agents, we employed a straightforward ball-milling process to mix DLS with highly conductive flake graphite. This process, conducted for 10 min, yielded a homogeneous composite powder, designated as GDLS. Scanning electron microscopy (SEM) imaging (Fig. S3) reveals the homogeneous mixing of rod-shaped DLS particles with flake graphite. The graphite flakes form interconnected pathways, constructing a percolating conductive network that ensnares the DLS particles, thereby enhancing the overall electronic conductivity of the composite. The first-cycle charge/discharge profiles of DLS and GDLS were compared within the voltage window of 3.0–4.4 V (Fig. S4). The intrinsically insulating nature of DLS impedes its electrochemical decomposition during charging, leading to a large overpotential and a limited charge capacity of only 260 mAh g^−1^. In stark contrast, the graphite-modified GDLS composite exhibits a significantly lower decomposition onset potential and delivers a markedly higher first-cycle charge capacity of 368 mAh g^−1^. This capacity remains substantially lower than the theoretical value (425 mAh g⁻^1^), which is partially attributed to the ongoing decomposition of DLS above 4.4 V. Half-cells were fabricated by incorporating 5 wt% of either DLS or the GDLS composite into the LCO cathode, denoted as DLS-LCO||Li and GDLS-LCO||Li, respectively. For comparison, a baseline LCO||Li cell was also prepared. A 4.4 V charge cutoff voltage was selected to prevent the excessive irreversible phase transitions and significant performance fade inherent to unmodified LCO at higher voltages (≥ 4.45 V), enabling a clear assessment of the minor prelithiation agent in its simple mixture with pristine LCO. All cells were activated for 3 cycles at 0.1 C before cycling at 1 C. The charge/discharge profiles of the first four cycles (Figs. S4 and S5) reveal that the DLS-LCO cell suffered from the insulating nature of DLS, exhibiting a noticeably lower discharge capacity than both the GDLS-LCO and the baseline LCO cells. Furthermore, its first-cycle charge plateau was significantly higher, indicating that the addition of unmodified DLS substantially increased the initial activation energy barrier for LCO. These results demonstrate that DLS acts as a "lithium consumer," deteriorating the performance of the LCO cathode. In contrast, the graphite-modified GDLS composite effectively functioned as a prelithiation agent ("lithium donor"): It enhanced the first-cycle charge capacity compared to the LCO||Li without compromising the discharge capacity, unlike DLS.

However, the amount of prelithiation agent is not "the more, the better." We compared the first-cycle profiles and the cycling performance over 100 cycles at 1 C for LCO half-cells containing 2, 5, and 8 wt% GDLS (Fig. S6). An excessive amount of GDLS (8 wt%) led to a noticeably elevated first-cycle charge plateau and a lower discharge capacity than the baseline LCO, mimicking the "blocking effect" observed with DLS. Although the cell with 2 wt% GDLS exhibited a lower first-cycle charge capacity than the 5 wt% group, its discharge capacity was unexpectedly higher. While this might suggest 2 wt% as the optimal loading, the lower charge capacity indicates less active lithium supplied to the system. Consequently, the 2 wt% cell showed the most rapid capacity fading over 100 cycles, even though its initial discharge capacity at 1 C was similar to the 5 wt% cell. Meanwhile, the 8 wt% cell delivered a lower overall capacity throughout cycling. Therefore, in this study, 5 wt% GDLS is preliminarily determined as the optimal addition amount, and this amount is used in subsequent comparative experiments and tests. The corresponding LCO with this addition amount is defined as GDLS-LCO.

**Fig. 1:**
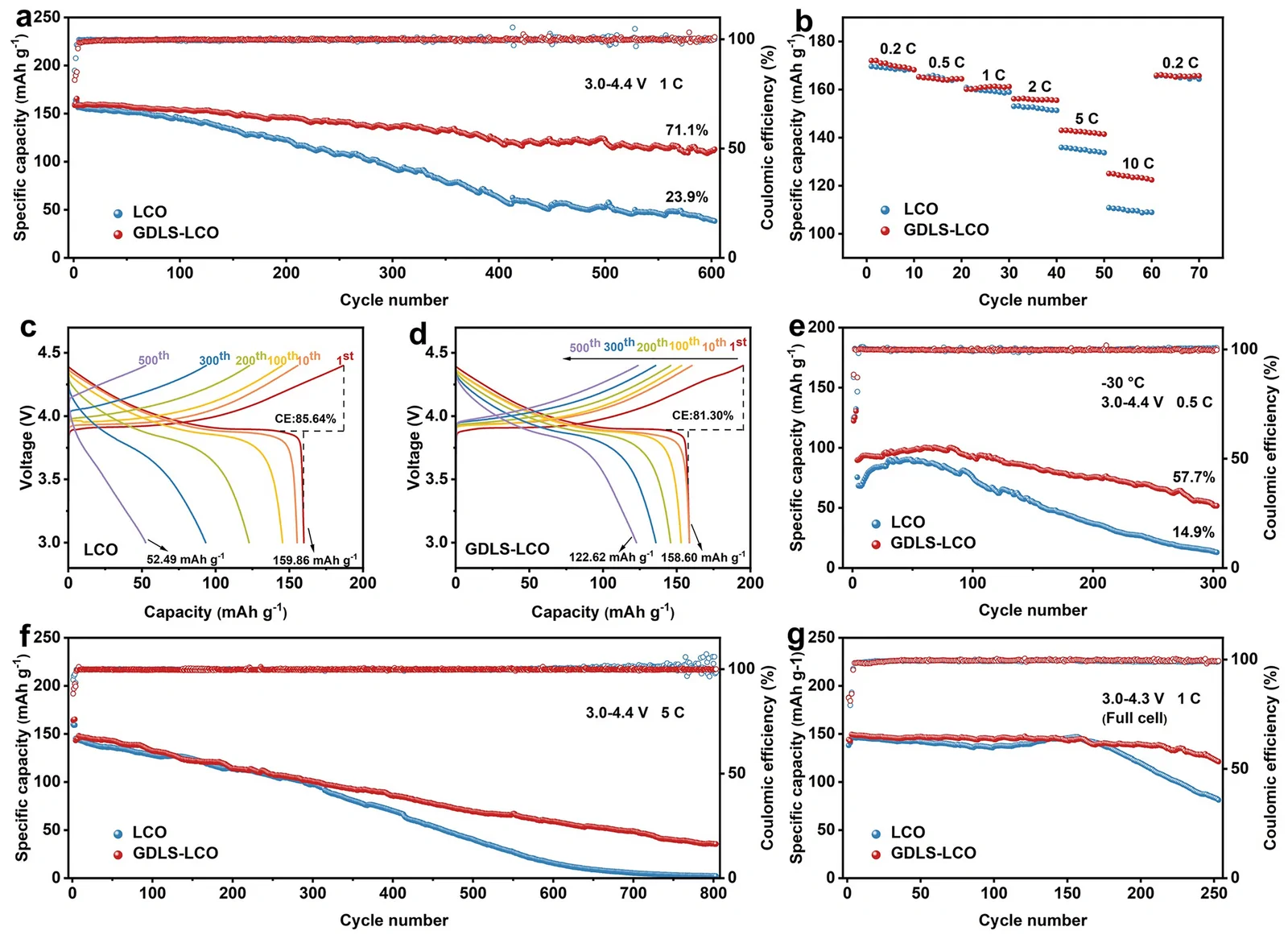
Electrochemical performance of LCO‖Li and GDLS-LCO‖Li half-cells within 3.0–4.4 V: **a** cycling performance at 1 C, **b** rate performance, **c, d** charge–discharge curves at 1 C, **e** cycling performance at 0.5 C and − 30 °C, and **f** cycling performance at 5 C. **g** Cycling performance of LCO‖graphite and GDLS-LCO‖graphite full cells within 3.0–4.3 V at 1 C

The cycling and rate capabilities of GDLS-LCO and LCO half-cells within 3.0–4.4 V were compared (Fig. 1a-f). The GDLS-LCO cell retained 71.1% of its capacity after 600 long-term cycles at 1 C at room temperature, significantly outperforming the LCO cell, which retained only 23.9%. At the 500th cycle, GDLS-LCO delivered a high capacity of 122 mAh g^−1^, vastly superior to the 52.49 mAh g^−1^ of the LCO cell at the same cycle number. Furthermore, the GDLS-LCO electrode demonstrated markedly enhanced rate capability, maintaining higher capacities, particularly at high rates (Fig. 1b). Further cycling tests at a high rate of 5 C for 800 cycles confirmed the superior capacity retention of GDLS-LCO over LCO (Fig. 1f), as anticipated. Since capacity retention at high rates is closely associated with ionic diffusion and charge transfer kinetics, we hypothesize that the GDLS-LCO electrode maintains higher Li⁺ diffusion coefficients and electronic conductivity throughout extended cycling. This hypothesis is corroborated by the low-temperature performance comparison (− 30 °C, Fig. 1e). Under these conditions, where microscopic kinetic processes are severely hampered, the difference between the two electrodes becomes even more pronounced [31]. The GDLS-LCO cell not only delivered a higher capacity but also exhibited slower capacity decay than the LCO cell. The enhanced performance was further validated in full cells configured with graphite anodes (GDLS-LCO||graphite vs. LCO||graphite) within a voltage range of 3.0–4.3 V. The GDLS-LCO||graphite full cell without offline prelithiation exhibits a high first-cycle Coulombic efficiency, which truly reflects the lithium-supplementing characteristic of the GDLS prelithiation agent (Fig. S7). The GDLS-LCO full cell effectively mitigated capacity fade and exhibited superior rate capability (Figs. 1g and S8). Moreover, even when the upper cutoff voltage was increased to 4.4 V, the GDLS-LCO full-cell maintained good cycling stability and rate performance (Fig. S9).

### Kinetic Enhancement

**Fig. 2:**
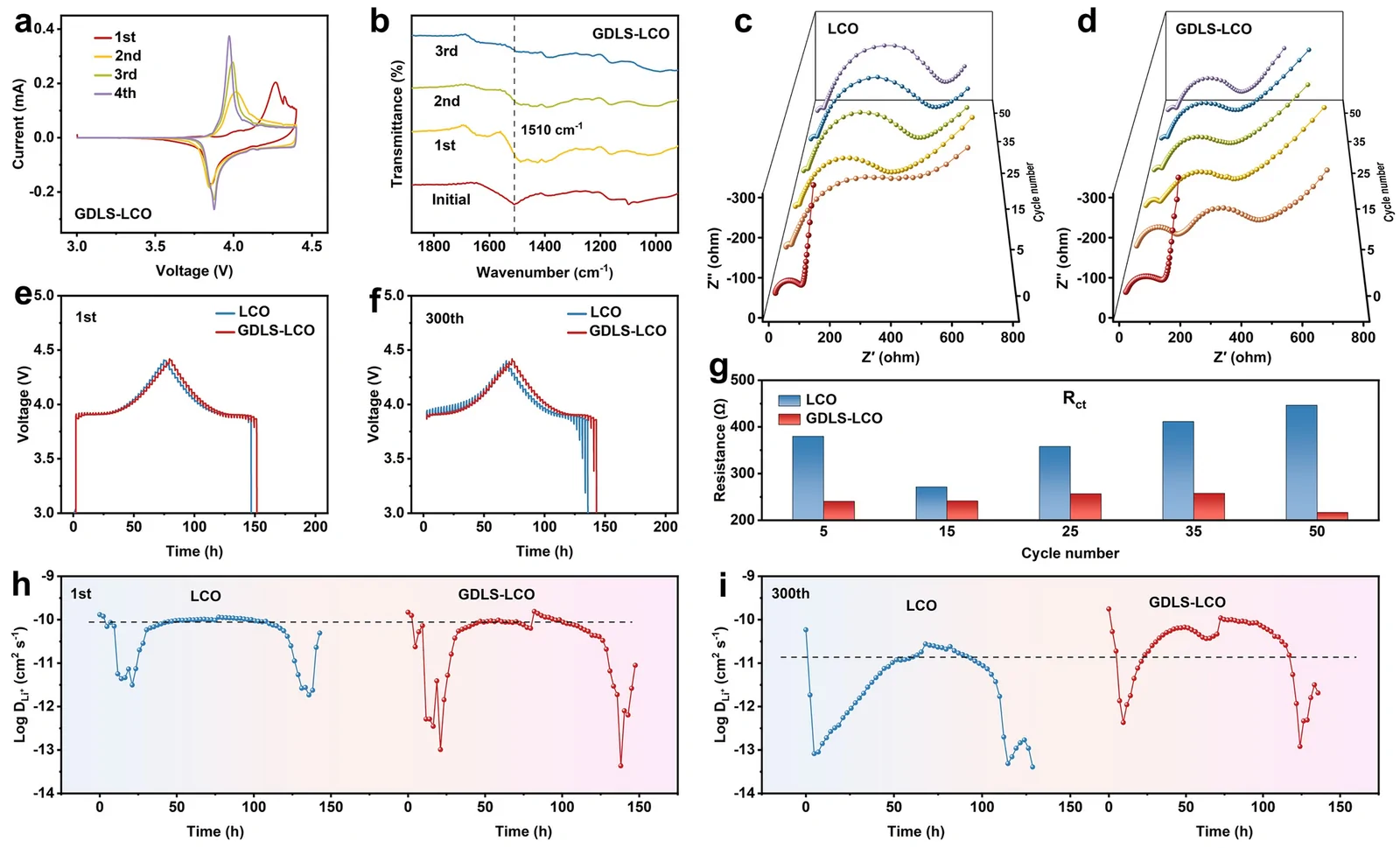
**a** CV curves of GDLS-LCO for the initial four cycles at a scan rate of 0.03 mV s^−1^. **b** FTIR spectra of the same GDLS-LCO electrode before cycling and after the first three cycles. **c, d** in situ EIS Nyquist plots for LCO and GDLS-LCO over the first 53 cycles (including 3 activation cycles). **e**, **f** GITT profiles for LCO and GDLS-LCO during the first cycle and the 300th cycle. **g** Corresponding R_ct_ values obtained from equivalent circuit fitting of the EIS data. **h**, **i** The values of Li^+^ diffusion coefficients calculated from the GITT

Notably, as seen in Fig. 1a, the incorporation of GDLS into LCO did not lead to a significant initial boost in discharge capacity, which is typical for conventional sacrificial cathode prelithiation agents. Instead, its primary benefit lies in its remarkable ability to suppress subsequent capacity fade. This suggests that the GDLS composite functions less as a mere "lithium donor" and more as a "cycle stabilizer" for the LCO cathode. An apparent discrepancy arises from the first-cycle charge capacities (Fig. S4): The DLS-LCO cell exhibited the highest value, which seems to contradict the earlier finding that GDLS itself delivers a higher capacity than DLS. This can be rationalized by two factors. First, the 5 wt% GDLS composite contains only ~ 3 wt% active DLS, meaning a lower absolute amount of the prelithiation agent was added. Second, we postulate that because LCO itself possesses a relatively high first-cycle Coulombic efficiency and its oxidation potential overlaps with that of the GDLS (Fig. S10), the GDLS incorporated into the cathode is not fully consumed in the first cycle. Instead, it is consumed gradually over multiple cycles. Although the initial oxidation onset potentials are similar, the positions of the oxidation peaks differ. The gradually evolving cyclic voltammetry (CV) profiles of GDLS-LCO over the first four cycles clearly show the sequential consumption of GDLS within the 3.0–4.4 V window at a scan rate of 0.03 mV s^−1^, as evidenced by the shifting oxidation peaks (Fig. 2a). To further corroborate this gradual consumption mechanism, Fourier transform infrared (FTIR) spectroscopy was performed on pristine GDLS-LCO and LCO electrodes (Fig. S11). The GDLS-LCO electrode exhibited a distinct absorption band at 1510 cm^−1^, absent in the LCO spectrum, which is attributable to the C=O stretching vibration of the squarate anion in DLS [32]. This assignment was confirmed by the strong corresponding band observed in the FTIR spectrum of the as-synthesized GDLS powder. Notably, a significant absorption peak at 1510 cm^−1^ persisted in the GDLS-LCO electrode even after the first cycle, confirming the incomplete consumption of GDLS. Subsequently, FTIR tests were performed on the same GDLS-LCO electrode after the first three cycles (Fig. 2b). The intensity of the 1510 cm^−1^ peak progressively diminished with cycling, becoming barely discernible only after the third cycle. This observation is fully consistent with the CV results. These findings indicate that the traditional self-sacrificing cathode lithium replenishing agent DLS, in GDLS-LCO, does not release all of its active lithium in the first cycle. Instead, it releases it gradually in a gentle manner during the initial electrochemical cycles. In subsequent gas evolution studies, it is clear that this gradual release is crucial for protecting the electrode morphology and structure.

As hypothesized earlier, the superior performance of GDLS-LCO likely stems from enhanced ionic diffusion and charge transfer kinetics. To verify this, a detailed kinetic analysis was conducted. In situ electrochemical impedance spectroscopy (EIS) measurements (Fig. 2c, d) revealed that the growth of interfacial impedance (indicated by the size of the semicircular arc) was significantly slower for the GDLS-LCO electrode compared to its LCO counterpart. The EIS spectra collected between the 8th and 53rd cycles were fitted using an appropriate equivalent circuit model (Fig. S12). The fitted charge transfer resistance (R_ct_), associated with the cathode–electrolyte interface and CEI growth, was consistently lower for GDLS-LCO throughout this period. Furthermore, its rate of increase was substantially suppressed compared to that of LCO (Fig. 2g). These results unambiguously demonstrate the more favorable interfacial kinetics of the GDLS-LCO electrode upon extended cycling. Additional cyclic voltammetry (CV) tests were performed at various scan rates (Fig. S13). The GDLS-LCO electrode exhibited smaller voltage polarization with increasing scan rates, indicative of more stable and rapid reaction kinetics. The galvanostatic intermittent titration technique (GITT) was employed to probe the Li^+^ diffusion coefficients for both cells in their first cycle and after 300 cycles at 1 C (Fig. 2e, f, h, i). Intriguingly, the calculated D_Li⁺_ for GDLS-LCO was initially lower than that for LCO in the first cycle [33], likely due to the incorporation of the less conductive DLS component. However, after 300 cycles, the situation was completely reversed, with the GDLS-LCO electrode exhibiting significantly higher Li^+^ diffusion coefficients overall. In summary, kinetic studies confirm that the incorporation of GDLS endows GDLS-LCO with superior Li⁺ diffusivity and charge transfer kinetics during long-term cycling, yet the underlying mechanism of GDLS's effects warrants in-depth exploration.

### Surface OVs Generation and CEI Thickness Modulation

**Fig. 3:**
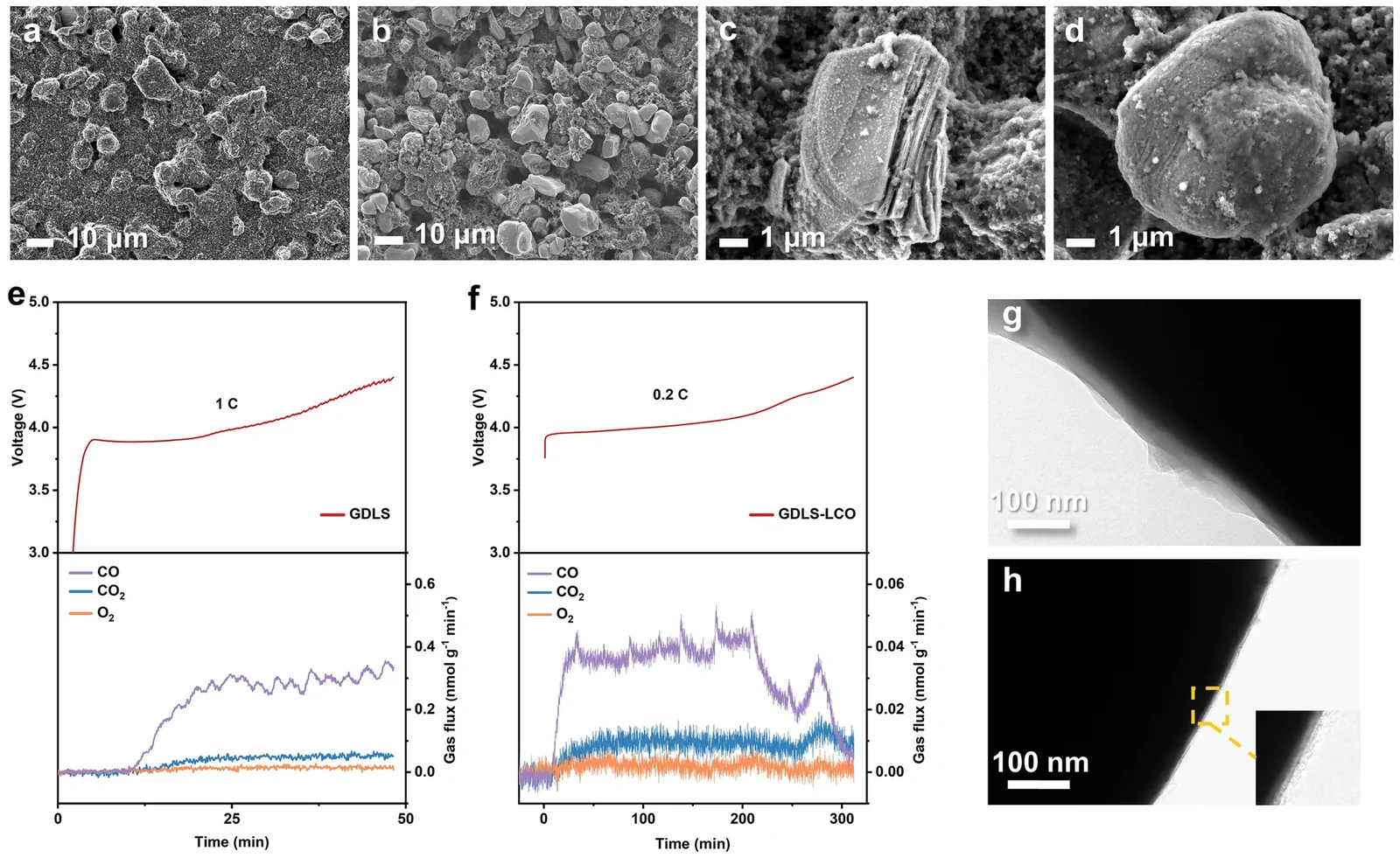
SEM images of **a** LCO and **b** GDLS-LCO electrodes after 3 cycles at 0.1 C. SEM images of LiCoO_2_ particles in **c** LCO and **d** GDLS-LCO electrodes after 500 cycles at 1 C. In situ DEMS analysis during the first charge process for **e** GDLS and **f** GDLS-LCO at 1 C. TEM images of CEI layers on **g** LCO and **h** GDLS-LCO electrodes after 300 cycles at 1 C

GDLS optimizes LCO either directly or indirectly, and the self-sacrificing nature of Li_2_C_4_O_4_ makes the latter more probable. Herein, we further explore its decomposition products and subsequent effects. Previous studies have established that DLS decomposition during cycling generates gaseous products, although the precise composition has been debated. Early reports suggested CO_2_ evolution [32, 34]. Shen et al. hypothesized the formation of C and CO_2_, proposing an initial cleavage of DLS into four identical ·C=O radicals (Li_2_C_4_O_4_ → 2Li^+^ + 2e^−^ + 4C = O), with these highly reactive radicals serving as intermediates for CO_2_ formation [34]. However, this pathway raises questions regarding electron counting balance, since ·C=O is a monoradical species. Based on the symmetric structure of the squarate dianion, we propose an alternative mechanism. The two carbon atoms far from the lithium atom each have two C–C bonds and the same chemical environment, while the chemical environments of the other two carbon atoms are also identical. Therefore, it is highly likely that the initial bond breakage results in the formation of $\dot{\cdot \mathrm{C}}=\mathrm{O}$ with two single-electron free radicals. This initial diradical intermediate, lacking a stable octet configuration, can undergo facile electronic rearrangement. This involves a change in hybridization from *sp*^2^ to *sp* (C≡O), leading to the formation of CO gas. While dimerization of radicals could potentially lead to CO_2_ and C, this pathway is likely less favorable. This mechanistic hypothesis is further corroborated by the subsequent work of Liu et al*.*, who employed in situ differential electrochemical mass spectrometry (DEMS) to convincingly demonstrate that the decomposition of a Li_2_C_4_O_4_/carbon nanotube composite (DLS-3CNT) produces CO as the primary gaseous product, with no other gases detected in significant quantities [35]. It should be noted that the decomposition products (CO_2_, CO, C) can interconvert under the complex local energy landscapes within a battery cell. Therefore, the exact gas composition may vary across different systems. In this work, DEMS was employed to analyze the GDLS composite (Fig. 3e). During the first charge at 1 C, the decomposition of GDLS primarily generated CO, with a small amount of CO_2_. An identical gas evolution profile was observed for the full GDLS-LCO cathode, confirming that the gaseous products in this system are predominantly CO derived from GDLS decomposition, with minor CO_2_. This result provides strong experimental validation for our proposed decomposition pathway.

As established earlier, GDLS is fully consumed by the third cycle. SEM imaging of the LCO and GDLS-LCO electrodes before cycling and after 3 cycles at 0.1 C (Figs. S13 and 3a, b) revealed, as expected, the formation of more numerous and pronounced pores in the cycled GDLS-LCO electrode. This observation provides direct morphological evidence for DLS decomposition and concomitant gas evolution. While violent gas evolution can undoubtedly damage the cathode morphology, the moderated decomposition of GDLS in this system, as previously discussed, results in a controlled formation of pores. As seen in Fig. 3b, this process effectively transforms the cathode into a more porous electrode, which facilitates electrolyte infiltration and enhances Li^+^ transport at the interface [36–38]. This porous structure, especially the large pores, can be maintained even after 600 cycles (Fig. S15). We posit that this improved ionic accessibility constitutes one contributing factor, among others, to the enhanced performance of the GDLS-LCO system. Energy-dispersive X-ray spectroscopy (EDX) analysis on the LCO particles within the cycled electrodes (Fig. S16) suggested a slightly higher surface O/Co ratio for GDLS-LCO. Recall that DLS decomposition produces $\dot{\cdot \mathrm{C}}=\mathrm{O}$ and CO gas, inspired by the work which demonstrated CO can adsorb lattice oxygen from CeO_2_ to form CO_2_, we hypothesized a similar surface reaction here. Given the reducing nature of CO and the strong oxidizing power of Co^4+^ on the delithiated LCO surface, we speculate that CO reacts with the LCO surface, oxidizing to CO_2_ while simultaneously reducing surface Co^4+^ and extracting lattice oxygen atoms, thereby generating OVs [39]. Furthermore, we postulate that the $\dot{\cdot \mathrm{C}}=\mathrm{O}$ is highly unstable. It may undergo a low-energy p*-p* electronic transition to form: C=O, without altering its hybridization. Since both p* orbitals containing a single electron are antibonding and symmetrical, the energy difference for this electron transition is low, making it highly likely to occur. Although the system will eventually tend to lower its energy and convert to CO, the intermediate state of: C=O remains significant. It is more stable than $\dot{\cdot \mathrm{C}}=\mathrm{O}$ and possesses an empty orbital, which acts as an electron pair acceptor. This Lewis acid structure facilitates its interaction with the oxygen atoms on the surface of LiCoO_2_ that expose electron pairs, leading to a Lewis acid–base neutralization reaction. Subsequently, it transforms into CO_2_, further refining the surface OVs profile. These two OV generation reactions were theoretically verified by the subsequent DFT calculations (Fig. 6g).

Since GDLS is simply mixed with LCO, the generated OVs are primarily concentrated on the LCO surface. Concurrently, some oxygen ions from the near-surface may migrate toward the surface to annihilate these vacancies, a process that in turn generates new OVs in the near-surface lattice. This results in a gradual penetration of OVs from the surface into a shallow subsurface region of the LCO particles. We propose that this gradient-rich OV-containing surface layer plays a crucial role in enhancing the electrochemical performance. Capacity degradation in LiCoO_2_ is typically accompanied by Co dissolution and microcracking due to layer sliding [40–44]. SEM images of the LiCoO_2_ particles after 500 cycles at 1 C (Fig. 3c, d) reveal severe cracking in the baseline LCO, whereas the GDLS-LCO particles maintain their structural integrity. This morphological observation is further corroborated by inductively coupled plasma mass spectrometry (ICP-MS) analysis of the electrolytes after 300 cycles (Fig. S17), which showed significantly less cobalt dissolution from the GDLS-LCO electrode. Furthermore, transmission electron microscopy (TEM) imaging (Fig. 3g, h) confirmed that the GDLS-LCO electrode was protected by a much thinner and more uniform CEI layer.

**Fig. 4:**
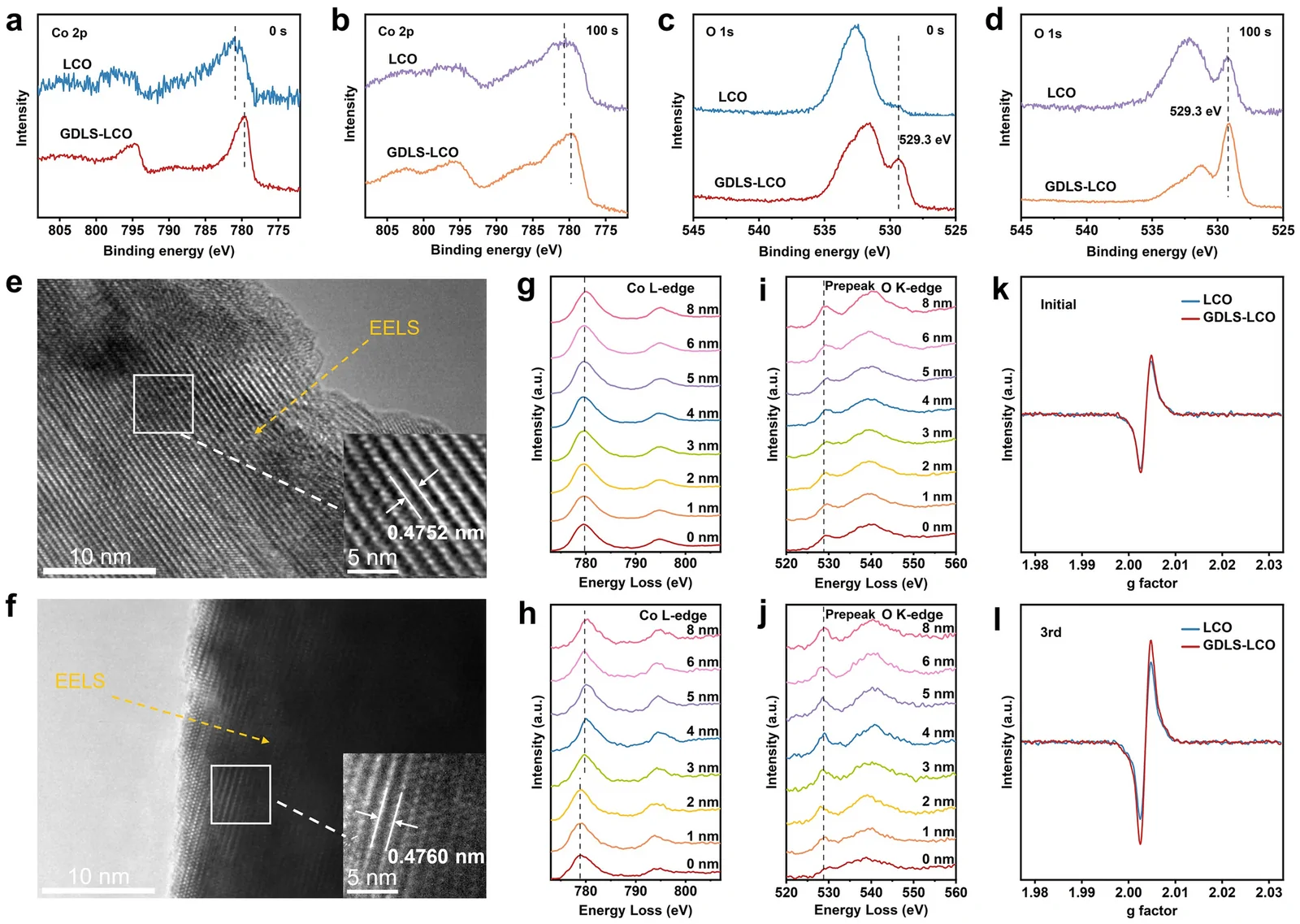
**a-d** XPS depth profiles of the LCO and GDLS-LCO electrodes after activation: XPS spectra of Co 2*p* and O 1*s* after Ar^+^ ion sputtering for 0 s and 100 s. **e-j** HRTEM images and corresponding near-surface EELS line scans (Co L-edge and O K-edge) of LiCoO_2_ particles in the activated LCO (**e, g, i**) and GDLS-LCO (**f, h, j**) electrodes. **k** EPR spectra of the LCO and GDLS-LCO electrodes in the initial state and after activation

The hypothesis that GDLS decomposition enriches the LCO surface with OVs requires direct evidence. To probe this, we conducted X-ray photoelectron spectroscopy (XPS) depth profiling on cathodes retrieved after three activation cycles, with Ar^+^ sputtering for 100 s to analyze subsurface regions (Fig. 4a-d). In the Co 2*p* spectra, the peaks for GDLS-LCO were shifted to lower binding energies, both at the surface and after sputtering, compared to LCO. This negative shift indicates a lower average oxidation state of cobalt in the near-surface region of GDLS-LCO, which is a direct consequence of OV formation. Each OV donates electrons to the surrounding lattice, reducing nearby Co^4+^ ions. The larger binding energy shift at the surface (0 s) compared to the sputtered region (100 s) for GDLS-LCO suggests a higher OV concentration at the very surface, consistent with a gradient distribution. The O 1*s* spectra show: The characteristic lattice oxygen (O^2−^) peak at 529 eV was markedly more intense for the GDLS-LCO electrode at 0s sputtering time. The attenuated signal for the LCO is attributed to masking by a thicker CEI layer. After 100s of sputtering, which removed the surface CEI, the lattice oxygen peak became visible for both electrodes but remained less intense for LCO, providing further confirmation of the thinner CEI on GDLS-LCO.

To gain further insight into the near-surface structure, the CEI layers were carefully removed from activated electrodes via ultrasonic treatment, followed by high-resolution TEM (HRTEM) and electron energy loss spectroscopy (EELS) analysis (Fig. 4e-j). HRTEM revealed clear lattice fringes corresponding to the (003) planes of LCO (d = 0.475–0.476 nm) in both samples [45]. EELS depth profiling across the top 0–2 nm, however, revealed critical differences: The Co L-edge for GDLS-LCO exhibited a shift toward lower energy loss, confirming a lower average Co oxidation state in its outermost surface layer [46]. This is a direct result of substantial lattice oxygen loss. Concurrently, the O K-edge of GDLS-LCO showed an attenuated pre-edge peak feature, consistent with the formation of surface OVs. These signature changes were absent in the LCO. The electrochemical cycling of GDLS-LCO generates a substantial quantity of OVs predominantly on the surface of the LiCoO_2_ particles, leading to an increase in the overall OV concentration within the cathode electrode. Electron paramagnetic resonance (EPR) spectroscopy of the full cathodes (including the Al current collector) before and after activation (Fig. 4k, l) reveals comparable initial OV signals for both samples, likely originating from intrinsic defects in the materials and the collector itself. After three cycles, the OV signal intensity increased for both electrodes, but the enhancement was markedly more pronounced for GDLS-LCO. The fact that the absolute difference is significant yet not overwhelmingly large precisely indicates that the additional OVs in GDLS-LCO are primarily concentrated at the surface rather than distributed throughout the bulk. This surface-specific OV generation is critically important: GDLS accelerates the selective removal of these unstable surface O^2−^ (weakly bonded oxygen atoms are preferentially removed) [11], thereby mitigating the further uncontrolled loss of lattice oxygen. The model proposed by Cai et al., which constructs an oxygen buffer layer through surface OVs, is also applicable to this study [13]. In subsequent cycles, EPR measurements and quantitative analysis performed on cathodes (without aluminum current collectors) confirm this. As shown in Fig. S18, the lattice oxygen loss of GDLS-LCO is mitigated during long-term cycling, exhibiting a lower OV content after 600 cycles (9.87 vs. 12.42 μmol g^−1^ for LCO). Furthermore, the formation of OVs is accompanied by the reduction of highly oxidative Co^4+^ species, thereby suppressing their accumulation. Continuous lattice oxygen loss during long-term cycling is inevitable. The EELS results after 600 cycles (Fig. S19) show that the bulk lattice oxygen in GDLS-LCO also undergoes gradual loss upon long-term cycling, which eventually causes the surface OVs to lose their specificity. Therefore, it is necessary to further understand how these OVs affect the battery cycling performance in the longer term. The accumulation of Co^4+^ is detrimental as it catalyzes the oxidative decomposition of the electrolyte, producing H_2_O and H^+^. These products trigger the hydrolysis of LiPF_6_, generating corrosive HF. HF etches the LCO surface, leading to cobalt dissolution, while the accumulation of LiPF_6_ decomposition products contributes to CEI thickening and overall performance degradation [20]. Therefore, the suppression of this cascade reaction by OV formation is a fundamental reason for the thinner CEI and superior cycling stability observed in GDLS-LCO. In summary, our findings demonstrate that the abundance of surface OVs can regulate the CEI thickness, with a higher surface OV concentration resulting in a thinner and more protective interphase layer.

### Regulation of CEI Composition by Surface OVs

**Fig. 5:**
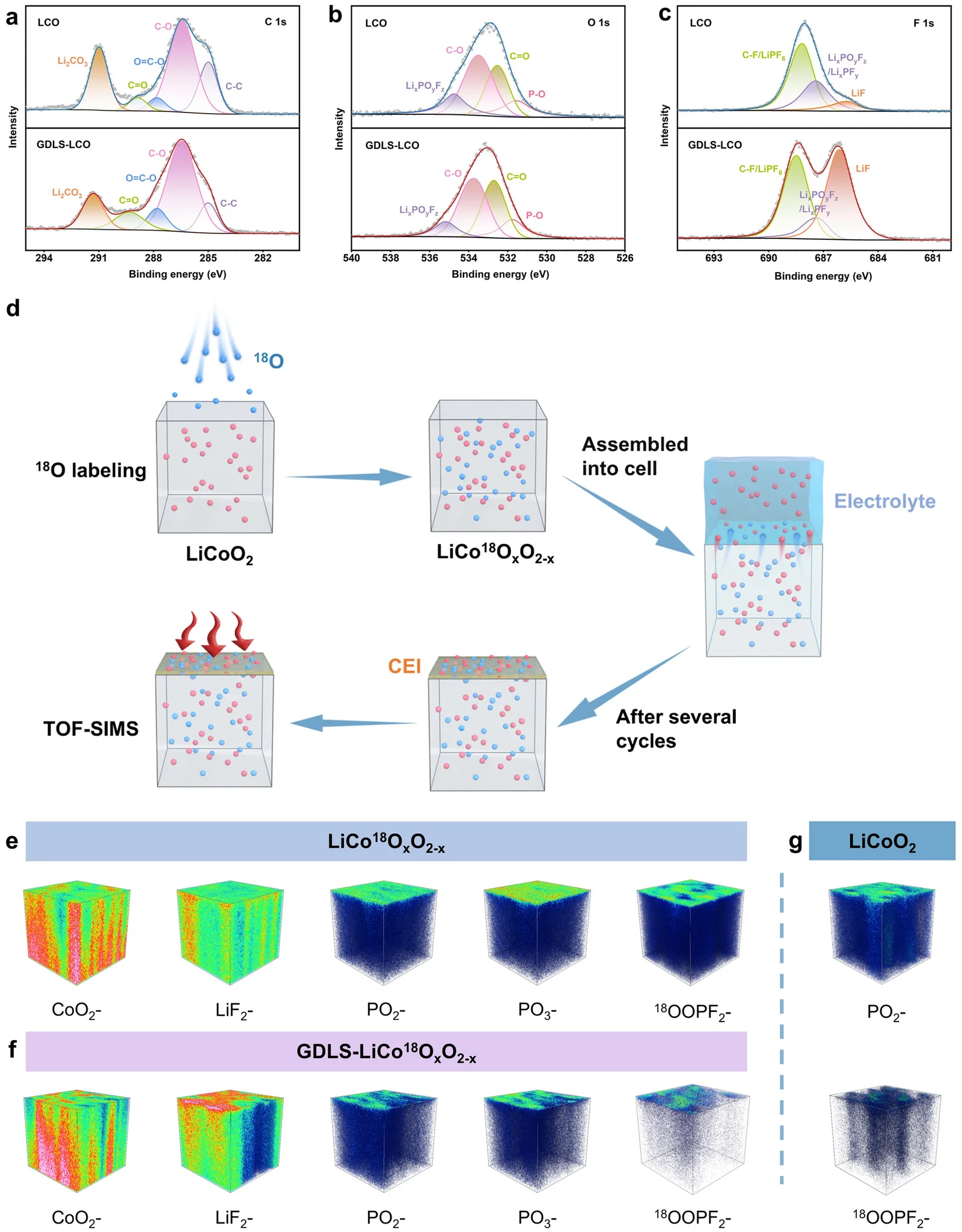
XPS spectra of the LCO and GDLS-LCO electrodes after 300 cycles: **a** C 1*s*, **b** O 1*s*, and **c** F 1*s* core-level regions. **d** Schematic illustration of the ^18^O isotope labeling combined with TOF–SIMS analysis. TOF–SIMS depth profiles of the secondary ion signals obtained from **e** LiCo^18^O_x_O_2-x_, **f** GDLS-LiCo^18^O_x_O_2-x_, and **g** LiCoO_2_ electrodes after 10 cycles

An OV-enriched surface not only suppresses the growth of the CEI but also fundamentally modulates its chemical composition. XPS survey spectra of cathode surfaces for LCO and GDLS-LCO after 300 cycles (Figs. S20 and 5a-c) show a more intense F 1*s* signal for the LCO, consistent with a thicker CEI layer, as fluorine originates solely from the decomposition products of LiPF_6_ and the electrolyte (e.g., fluoroethylene carbonate, FEC). LiPF_6_ decomposition primarily yields LiF and Li_x_PO_y_F_z_ species. A summary of the XPS peak positions (C 1*s*, O 1*s*, F 1*s*) and their corresponding compound ratios is listed in Tables S2-S4. The F 1*s* spectra (Fig. 5c) indicate a higher relative proportion of LiF in the CEI on GDLS-LCO, whereas the CEI on LCO is richer in Li_x_PO_y_F_z_, a finding corroborated by the O 1*s* spectra (Fig. 5b). Early studies attributed the oxygen in Li_x_PO_y_F_z_ primarily to H_2_O from electrolyte decomposition [20]. It was later hypothesized that surface lattice oxygen also participates extensively in reactions with PF_6_^−^ to form Li_x_PO_y_F_z_ and HF, a process often accompanied by ethylene carbonate (EC) ring-opening oligomerization, introducing detrimental organic oligomers into the CEI [19, 24]. This is consistent with the stronger C–C signal from oligomerized EC in the C 1*s* spectrum of LCO. Thus, an increased Li_x_PO_y_F_z_ content often signifies a higher abundance of organic species within the CEI. However, the oxygen source had not been experimentally verified. To directly trace the origin of oxygen, we synthesized ^18^O-isotope-labeled LiCo^18^O_x_O_2-x_, where a portion of the lattice oxygen was replaced using H_2_^18^O. As shown in Fig. 5d, if the hypothesis is correct, significant ^18^O enrichment should be detected in the CEI. Time-of-flight secondary ion mass spectrometry (TOF–SIMS) depth profiling of the cycled electrodes (covering the CEI and the outer LCO layer) was performed after 10 cycles. To exclude the background from natural abundance ^18^O, we compared the unlabeled LCO with the ^18^O-labeled LiCo^18^O_x_O_2-x_ (Fig. 5e, g). A dramatically more intense ^18^OOPF_2_-secondary ion signal was detected in the CEI of the labeled sample, unambiguously proving that lattice oxygen from LCO is a major source of oxygen in Li_x_PO_y_F_z_ and other oxygen-containing decomposition products. Since LiPF_6_ cannot penetrate the bulk lattice, the surface OV concentration undoubtedly dictates the extent of Li_x_PO_y_F_z_ formation. TOF–SIMS results for LiCo^18^O_x_O_2-x_ and GDLS-LiCo^18^O_x_O_2-x_ (Fig. 5e, f) also show a significantly stronger LiF_2_- signal for GDLS-LiCo^18^O_x_O_2-x_, indicating a higher LiF content. In contrast, the CEI on LiCo^18^O_x_O_2-x_ exhibits more intense PO_2_-, PO_3_-, and ^18^OOPF_2_-signals, confirming its higher content of oxygen-containing decomposition products like Li_x_PO_y_F_z_. A CEI rich in LiF is desirable as it forms a dense, corrosion-resistant, and ionically conductive layer that protects the electrode and facilitates interfacial kinetics [47]. Conversely, Li_x_PO_y_F_z_ species offer inferior mechanical robustness and ionic conductivity, and their formation is often accompanied by harmful organic oligomers and HF. In conclusion, we demonstrate that engineering the surface OVs concentration is an effective strategy to tailor CEI composition. A higher OV abundance promotes the formation of a beneficial LiF-rich CEI, resulting in a lower Li_x_PO_y_F_z_/LiF ratio.

### Theoretical Validation of the Comprehensive Regulation Mechanism

The decomposition pathway of Li_2_C_4_O_4_ and its regulatory mechanism on the formation of OVs on the LCO surface were further elucidated using density functional theory (DFT) calculations.

**Fig. 6:**
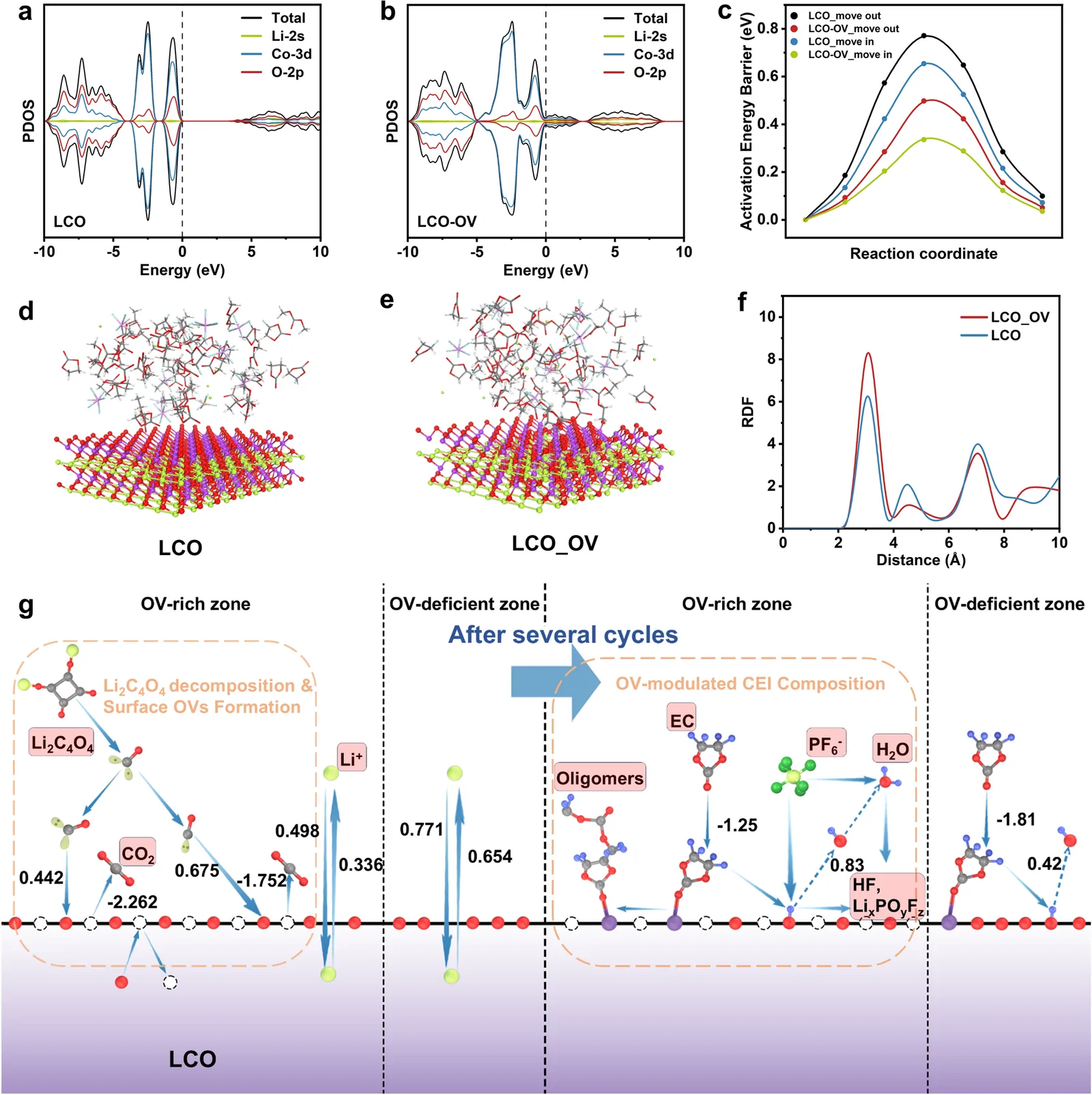
PDOS for LCO **a** without and **b** with OVs. **c** Comparison of the Li⁺ migration energy barriers across the LCO surface with and without OVs. **d-f** Solvation kinetics and radial distribution functions of LCO and LCO-OV. **g** Schematic illustrations and calculated energy barriers (in eV) for relevant reactions, including Li_2_C_4_O_4_ decomposition, surface OVs formation, and the OV-modulated CEI composition process

Energy evolution analysis reveals that the initially generated $\dot{\cdot \mathrm{C}}=\mathrm{O}$ from Li_2_C_4_O_4_ decomposition tends to undergo structural rearrangement to form the more stable: C=O intermediate and CO molecule, with CO exhibiting the lowest energy state (Fig. S21). The: C=O intermediate, characterized by its unique Lewis acid structure, demonstrates a lower adsorption energy barrier of 0.442 eV compared to CO (0.675 eV). Furthermore, its subsequent desorption as CO₂ is energetically more favorable, making it more effective in generating surface OVs (Figs. 6g and S22). These results confirm that both: C=O and CO can directly interact with the LCO surface to create OVs.

Surface OVs significantly modulate interfacial kinetics. As shown in Fig. 6a, b, the O 2*p* band center shifts to lower energy levels in OV-rich LCO (LCO-OV), which suppresses further oxygen release and stabilizes the lattice oxygen. Concurrently, the Co 3d band center moves closer to the Fermi level, reducing electron localization and enhancing electronic conductivity. Electron localization function (ELF) analysis (Fig. S23) clearly shows expanded regions of similar ELF values around cobalt atoms adjacent to OVs in LCO-OV. These comparable ELF values indicate similar local kinetic energy distributions, suggesting increased electron delocalization and consequently faster electron migration rates in LCO-OV. Additionally, the energy barriers for Li^+^ migration into and out of the LCO surface along the ab-plane (Fig. 6c and migration pathways in Fig. S24) are reduced in the presence of OVs [48], facilitating more efficient Li^+^ transport. Thus, a higher concentration of surface OVs enhances both electron mobility and Li⁺ diffusion kinetics at the LCO interface.

Finally, surface OVs effectively regulate the thickness and composition of the CEI. As shown in Fig. 6g, EC adsorption on the OV-rich LCO surface is less energetically favorable, and the subsequent ring-opening reaction requires overcoming a higher energy barrier (Fig. S25). This suppresses EC adsorption and ring-opening oligomerization, preventing excessive CEI growth and organic species accumulation. Lattice oxygen on the surface can react with PF_6_^−^ through two pathways to form Li_x_PO_y_F_z_ and HF: One is reacting with surface hydroxyl groups, and the other is reacting with H_2_O generated from the desorption of hydroxyl groups from the LCO surface. Both pathways require the presence of surface hydroxyl groups, most of which come from the combination of H and surface oxygen atoms after EC adsorption. Due to reduced EC adsorption and fewer available oxygen sites for hydrogen binding on OV-rich surfaces, the concentration of surface hydroxyl groups is significantly diminished. Furthermore, calculations show that the desorption energy barrier for surface hydroxyl groups increases in the presence of OVs, making water formation and subsequent reaction with PF_6_^−^ more difficult (Fig. S26), thereby inhibiting Li_x_PO_y_F_z_ generation. Consequently, an OV-enriched surface promotes the formation of a thinner CEI with reduced Li_x_PO_y_F_z_ content. The solvation kinetics and radial distribution functions of LCO and LCO-OV verify this process. As shown in Fig. 6d–f, the molecular arrangement on the LCO-OV surface differs from that on LCO with a larger distance span. The LCO-OV peak is significantly higher at 2–4 Å, indicating denser short-range molecular distribution, corresponding to the higher order and compactness of LiF relative to Li_x_PO_y_F_z_, while the lower LCO peak accounts for the mixture of Li_x_PO_y_F_z_ and oligomers. LCO-OV shows lower values at 4–10 Å, suggesting a looser medium-range structure, which optimizes the mechanical rigidity of CEI and prevents LCO cracking during volume changes caused by excessive LiF growth.

Theoretical calculations confirm that:

Decomposition products of Li_2_C_4_O_4_ can effectively generate surface OVs through two plausible pathways.

A high concentration of surface OVs facilitates electron migration and promotes Li⁺ transport across the interface.

An OV-rich surface enables the formation of a thinner, more superior CEI layer with minimized Li_x_PO_y_F_z_ content, contributing to improved electrochemical performance.

Theoretical calculation results further demonstrate the systematic regulation of interfacial kinetics and CEI layer properties by surface OVs.

### Multi-Dimensional Regulation of Surface OVs on CEI and Feasibility Verification

**Fig. 7:**
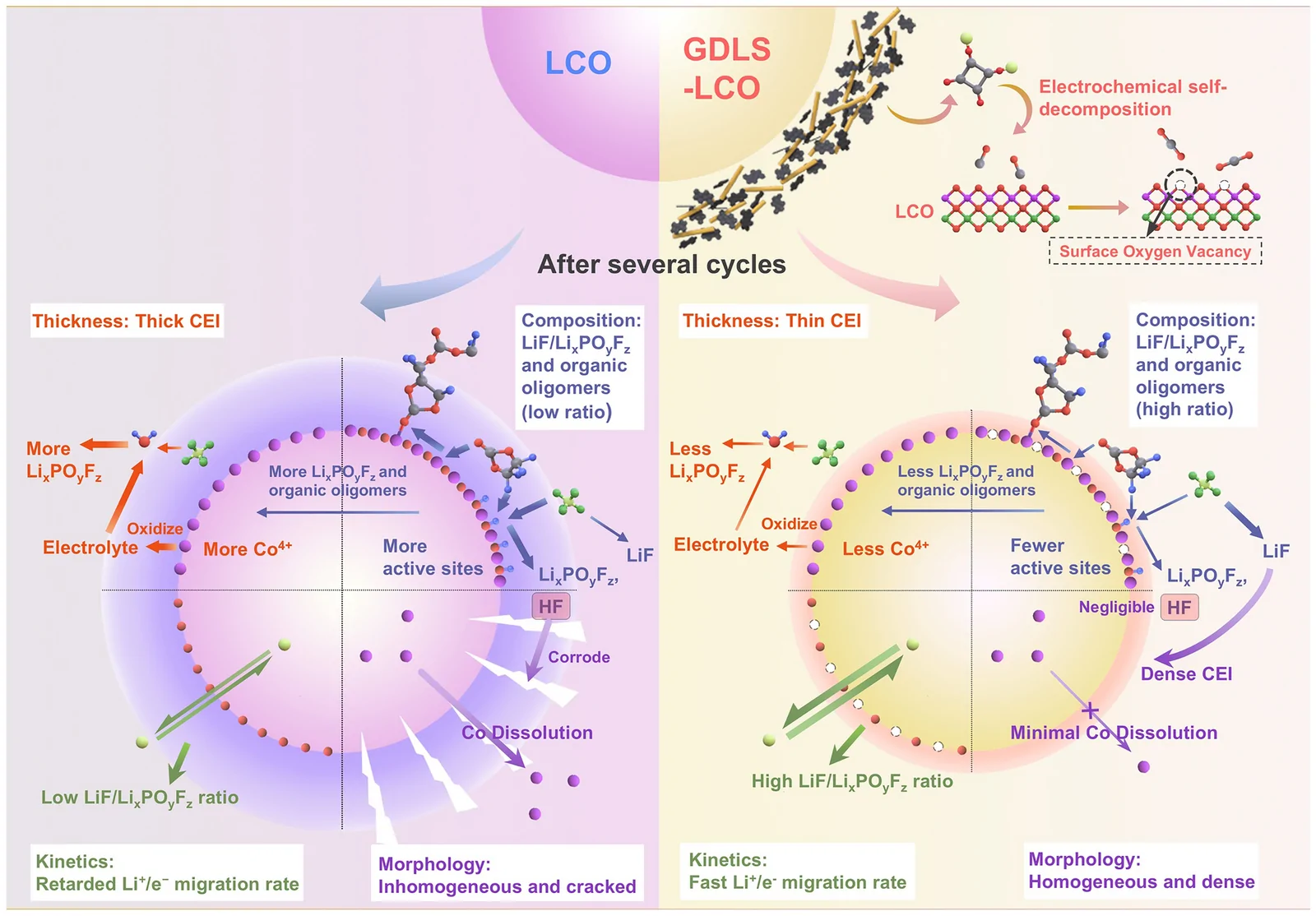
Schematic illustration of the multifaceted CEI regulation by surface OVs

Surface OV engineering regulates the CEI across four dimensions: composition, thickness, morphology, and kinetics (Fig. 7).

Composition: Increased surface OVs reduce the available lattice oxygen sites for LiPF_6_ and EC adsorption/reaction, thereby significantly suppressing the generation of Li_x_PO_y_F_z_, organic oligomers, and HF. Consequently, the CEI becomes richer in LiF, the other major decomposition product of LiPF_6_.

Thickness: The suppression of Li_x_PO_y_F_z_ and oligomer formation slows CEI thickening. Furthermore, surface OVs lower the average oxidation state of transition metals (TMs), reducing Co^4+^ content. This mitigates Co^4+^-catalyzed electrolyte oxidation (which produces water that reacts with LiPF_6_ to form more Li_x_PO_y_F_z_), further limiting the accumulation of detrimental CEI products and reducing overall thickness.

Morphology: HF, generated alongside Li_x_PO_y_F_z_, corrodes both the CEI and LCO, leading to crack formation and accelerated cobalt dissolution. The OV-modified surface minimizes HF production. Coupled with its higher relative LiF content, this promotes the formation of a uniform and dense CEI layer.

Kinetics: Calculations indicate that surface OVs lower the energy barrier for Li⁺ transport across the interface. Additionally, LiF offers superior Li⁺ conductivity compared to Li_x_PO_y_F_z_. Therefore, the high LiF/Li_x_PO_y_F_z_ ratio in the OV-engineered CEI results in significantly enhanced interfacial kinetics.

This strategy was applied to pouch cells, which are more consistent with practical application scenarios. For DLS that generates gas via first-cycle decomposition, no additional gas evolution occurs after the cell undergoes activation and degassing prior to formal tests (Fig. S27). Both the GDLS-LCO‖Li and GDLS-LCO‖graphite pouch cells exhibited excellent capacity and cycling stability (Fig. S28); in particular, the GDLS-LCO‖Li pouch cell delivered a capacity of 1 Ah with stable cycling performance, demonstrating the feasibility of this strategy for practical applications.

**Fig. 8:**
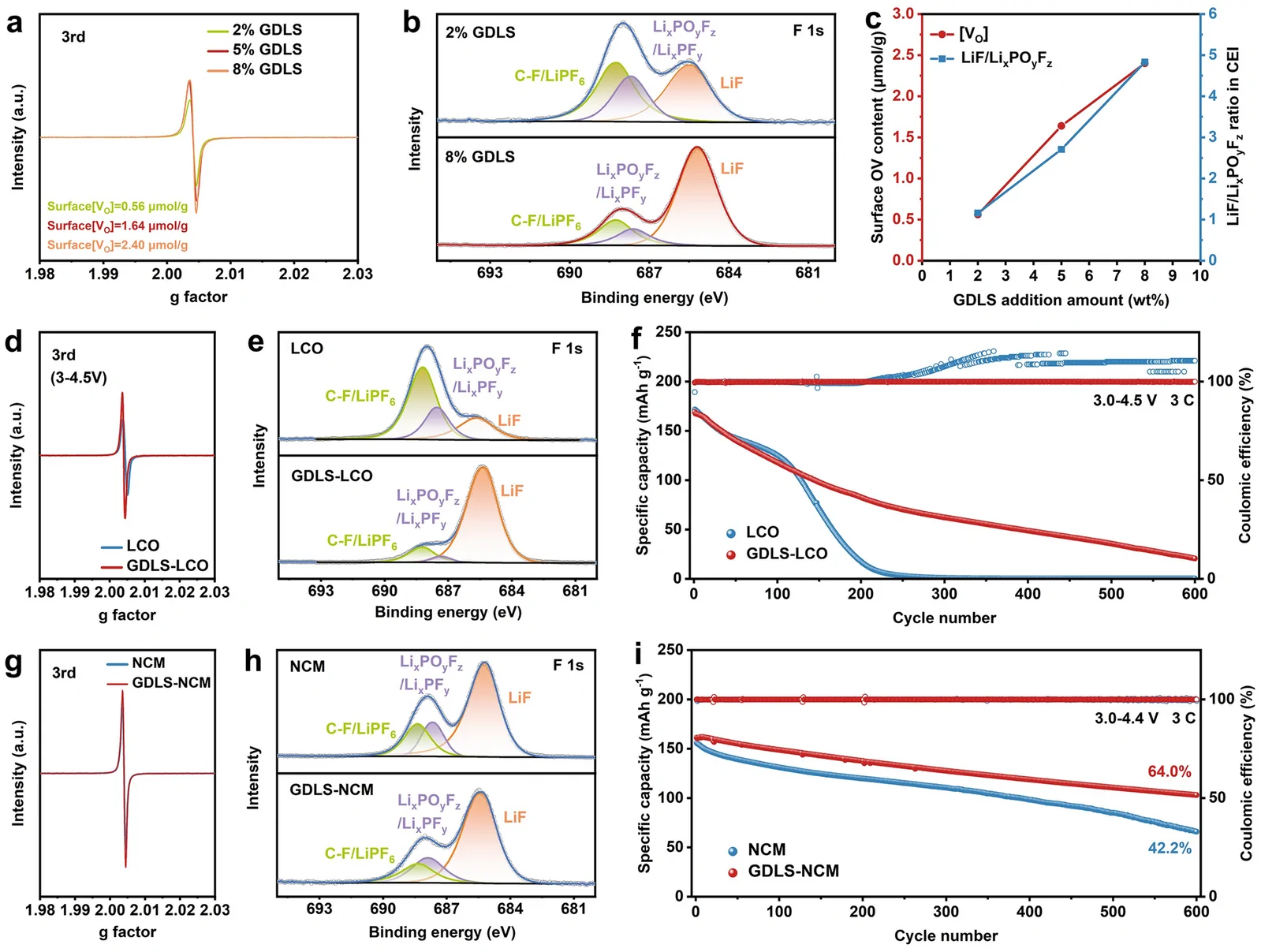
GDLS-LCO with different GDLS doping contents: **a** EPR spectra and quantitative analysis of activated cathodes, **b** F 1*s* core-level XPS spectra after 300 cycles, **c** linear relationships among GDLS doping content, surface OV concentration, and LiF/Li_x_PO_y_F_z_ ratio in the CEI layer. Feasibility analysis at 4.5 V for LCO and GDLS-LCO: **d** EPR spectra of activated cathodes, **e** F 1*s* core-level XPS spectra after 300 cycles, **f** cycling performance at 3C. Feasibility analysis for NCM811 and GDLS-NCM811: **g** EPR spectra of activated cathodes, **h** F 1*s* core-level XPS spectra after 300 cycles, **i** cycling performance at 3 C

To explore the feasibility of achieving relative quantification with this regulation strategy, GDLS-LCO cathodes with three different GDLS doping contents (2, 5, and 8 wt%) were fabricated and characterized. In Fig. 8a, EPR measurements were performed on the activated cathodes, and the surface OV concentrations were calculated using the same method as for pristine LCO (Fig. S18). The results indicated that the surface OV concentrations of the three groups increased with the rise in GDLS doping content. In addition, XPS measurements were conducted on the cells with 2 and 8 wt% GDLS after 300 cycles (Fig. 8b). The peak intensity ratio of the F 1s signal can reflect the content ratio of components in the CEI layer to a certain extent. The obtained LiF/ Li_x_PO_y_F_z_ ratios and corresponding surface OV contents are both presented in Fig. 8c, from which a distinct positive linear correlation between them is clearly observed. A definitive conclusion can be drawn that within a certain concentration range, the surface OV concentration of LCO increases with the increasing GDLS doping content, thereby regulating the elevation of the LiF/ Li_x_PO_y_F_z_ ratio, and vice versa. This conclusion verifies the feasibility of realizing relative quantification via the surface OV regulation strategy.

Meanwhile, to broaden and verify the universal applicability of this strategy, EPR measurements, XPS analyses, TEM characterizations, and cycling performance tests at 3 C current density were conducted on the activated LCO cathodes under the 4.5 V high-voltage scenario (Fig. 8d-f) and LiNi_0.8_Co_0.1_Mn_0.1_O_2_ (NCM811) cathodes (Fig. 8g-i), respectively. The results demonstrated that all cells modified with GDLS possessed higher OV concentrations after activation, and the formed CEI layer was thinner with a more optimized composition (Figs. S29 and S30), thus leading to enhanced cycling stability. At 4.5 V, pristine LCO nearly lost its electrochemical activity after 200 cycles, while GDLS-modified LCO still retained a considerable capacity. In the meantime, GDLS-modified NCM811 maintained a capacity retention of 64.0% after 600 cycles, which was significantly higher than that of pristine NCM811 (42.2%). These results fully demonstrate the great practical application potential of this strategy for diverse application scenarios.

## Conclusion

In summary, we systematically elucidate the pivotal role of cathode surface OVs in modulating interfacial kinetics and the cathode–electrolyte interphase. This work first reports that the incorporation of Li_2_C_4_O_4_ into LCO triggers its spontaneous decomposition during electrochemical cycling, leading to the formation of an OV-rich surface layer. The modified LCO exhibits significantly improved cycling stability (71.1% capacity retention after 600 cycles at 1 C vs. 23.9% for pristine LCO), along with enhanced rate capability and low-temperature performance at − 30 °C. A detailed decomposition pathway for DLS is proposed, and the mechanism of OV formation is rationalized through first-principles calculations. Experimental and theoretical studies reveal that this OV-enriched surface significantly enhances the interfacial kinetics of LCO. Most importantly, we decipher the mechanistic underpinnings of how surface OVs regulate the CEI: (1) OVs reduce the average oxidation state of surface cobalt, suppressing Co^4+^ accumulation This mitigation inhibits the Co^4+^-catalyzed oxidative decomposition of the electrolyte, thereby curbing the excessive growth of the CEI layer. (2) Employing ^18^O labeling combined with TOF–SIMS, we show that LCO lattice oxygen is a major oxygen source in LiPF_6_-derived Li_x_PO_y_F_z_ species, which form alongside harmful organics and HF. DFT calculations also confirm OV-rich surfaces increase Li_x_PO_y_F_z_ formation barrier and EC’s ring-opening barrier. Consequently, these promote a LiF (the other decomposition product of LiPF_6_)-rich CEI with superior ionic conductivity and mechanical robustness. This regulation mechanism is found to be equally applicable under 4.5 V high voltage and for NCM811 cathode. Therefore, engineering the surface OV concentration emerges as a powerful strategy for concurrently tailoring both the thickness and the chemical composition of the CEI. This insight provides a fundamental methodology and theoretical framework for the rational design of durable Li-ion batteries.

## Supplementary Information

Below is the link to the electronic supplementary material.

Supplementary file1 (DOCX 24267 KB)
