# Supplementary material for: Unlocking Electrochemical-Driven Surface Oxygen Vacancies-Regulated Cathode–Electrolyte Interphase for Stabilizing Li-Ion Cells
Source: Nanomicro Lett. 2026 May 9;18:361. doi: 10.1007/s40820-026-02170-3 (PMC13156354; doi:10.1007/s40820-026-02170-3)
Supplement: Supplementary file 1 — Supplementary file1 (DOCX 24267 KB) [file 40820_2026_2170_MOESM1_ESM.docx]

Supporting Information for

**Unlocking Electrochemical Driven Surface Oxygen Vacancies Regulated Cathode-Electrolyte Interphase for Stabilizing Li-ion Cells**

Chenxi Yan^1,2^, Xing Liu^2^, Xuanlong He^2^, Junjie Han^2^, Longjun He^1^, Na Tian^2^, Yanyi Wang^1^, Qiang Li^1^, Ning Zhao^3,^*, Lipeng Zhang^2,^*, Peixin Zhang^1^, Dingtao Ma^1,^*

^1^ College of Chemistry and Environmental Engineering, Shenzhen University, Shenzhen 518060, P. R. China

^2^ School of Materials and New Energy, South China Normal University, Shanwei 516600, P. R. China

^3^ College of Materials and Environmental Engineering, Shenzhen Polytechnic University, Shenzhen 518055, P. R. China

*Corresponding authors. E-mail: [zhaoning@szpu.edu.cn](mailto:zhaoning@szpu.edu.cn) (Ning Zhao ); [20219096@m.scnu.edu.cn](mailto:20219096@m.scnu.edu.cn) (Lipeng Zhang); [mdt25mdt2500@szu.edu.cn](mailto:mdt25mdt2500@szu.edu.cn) (Dingtao Ma)

**S1 Experimental Section**

**Material**

H_2_C_4_O_4_ (≥98%, Aladdin), Li_2_CO_3_ (99.5%, Macklin), graphite (≥98%, Aladdin), H_2_^18^O (≥97 atom% ^18^O, Aladdin), Co_3_O_4_ powder (≥99.9%, Aladdin), Lithium-ion battery electrolyte (1 M LiPF_6_ in DEC:EC:EMC = 1:1:1 by volume with 5% FEC)，Lithium metal foils were purchased from Dongguan CANRD New Energy Technology Co., Ltd. All the above materials were used directly without further purification.

**Material synthesis**

Synthesis of DLS and GDLS: H_2_C_4_O_4_ and Li_2_CO_3_ were employed as starting materials at a molar ratio of 1.03:1, with H_2_C_4_O_4_ in slight excess. Initially, H_2_C_4_O_4_ was added to and dissolved in deionized water at 60 °C under stirring until a colorless, transparent solution was obtained. After 10 minutes, the pre-ground Li_2_CO_3_ powder was introduced into the solution. The mixture was maintained at 60 °C with continuous stirring for 1 h. Subsequently, the solution was transferred to an oven and dried at 80 °C. The resulting solid was washed with anhydrous ethanol and then dried again at 80 °C to yield the final DLS powder. The GDLS composite was prepared by mechanically mixing the as-synthesized DLS with flake graphite. The precursors (DLS and graphite at a mass ratio of 3:2) were first individually ground and then subjected to a high-speed ball-milling process for 10 minutes to achieve a homogeneous mixture (GDLS).

Synthesis of LiCo^18^O_x_O_2-x_: A mixture of H₂¹⁸O and deionized water (volume ratio 1:4) was first prepared. Lithium metal foils were added cautiously to this mixture (piece by piece to mitigate the vigorous reaction) until the reaction was completed. Excess lithium metal was then removed. CO_2_ gas was bubbled through the resulting solution until a thick white suspension formed and no further thickening was observed. The suspension was dried at 80 °C to obtain a solid powder, which was subsequently washed with anhydrous ethanol and dried again at 80 °C to yield Li_2_C^18^O_x_O_3-x_. This intermediate product was mixed with Co_3_O_4_ at a stoichiometric ratio of Li/Co = 1.03, followed by thorough grinding. The blended powder was heated in air at a ramp rate of 3 °C/min to 900 °C, held at this temperature for 15 h, and finally ground to obtain the target material LiCo^18^O_x_O_2-x_.

**Materials Characterizations**

The surface morphology and elemental distribution were characterized by field emission scanning electron microscopy (FE-SEM) (JEOL, JSM-7800F) equipped with an energy-dispersive X-ray spectroscopy (EDS) detector (Ametek, TEAMOctane Plus) operating at 15 kV. Transmission electron microscopy (TEM) was carried out on a JEM-2100 & X-Max80 instrument at an acceleration voltage of 200 kV. X-ray diffraction (XRD) patterns were collected on a PANalytical Empyrean diffractometer with Cu Kα radiation (λ = 1.54065 Å), using a generator setting of 45 kV and 40 mA. Fourier-transform infrared (FTIR) spectra were acquired on a Thermo Fisher Nicolet iS50 spectrometer. In situ differential electrochemical mass spectrometry (DEMS) was performed using a GAS100-Li system (Shanghai Linglu Instrument Equipment Co., Ltd.). X-ray photoelectron spectroscopy (XPS) measurements were conducted on a Thermo Fisher Scientific K-Alpha instrument with Al Kα radiation, using a step size of 0.05 eV. High-resolution TEM (HRTEM) imaging and electron energy loss spectroscopy (EELS) line scans were obtained on an FEI Tecnai G2 F30 microscope. Electron paramagnetic resonance (EPR) spectroscopy was performed on a Bruker E500 spectrometer. Time-of-flight secondary ion mass spectrometry (TOF-SIMS) 3D reconstructions were generated using an ION TOF-SIMS 5 system.

**Electrochemical performance measurements**

The cathode slurry was prepared by uniformly mixing active material, acetylene black, and PVDF at a mass ratio of 8:1:1 (for DLS||Li and GDLS||Li cells, the ratio was adjusted to 6.5:2.5:1) in N-methyl-2-pyrrolidone (NMP), followed by coating onto aluminum foil and vacuum-drying at 80 °C for 10 hours. CR2032-type coin cells were used for all battery assembly and electrochemical tests. The material loading was controlled within 1.5-2.0 mg/cm². For full cells, the anode was fabricated using graphite, acetylene black, and PVDF at a mass ratio of 8:1:1, coated onto copper foil with procedures identical to those of the cathode. The negative/positive (N/P) ratio was designed between 1.05 and 1.15. All cells were assembled in an argon-filled glove box with H₂O and O₂ levels below 0.01 ppm. During testing, the 1 C current density was defined as 425 mAh g^-1^ for DLS electrodes, and 170 mAh g^-1^ for LCO and GDLS-LCO electrodes. All cells underwent activation by three cycles at 0.1 C. Polypropylene (PP) membrane was used as the separator in all configurations. For full-cell assembly, the graphite anode was prelithiated before cycling. Specifically, the graphite electrode was first assembled into a half-cell and cycled three times at 0.1 C (1 C = 350 mAh g^-1^) after which it was extracted and used to construct the full cell. All charge-discharge tests, except those conducted at -30 °C, were performed at room temperature (25 °C) using a LAND CT3001A battery test system. The galvanostatic intermittent titration technique (GITT) tests were performed using a 20 min titration step at 0.1 C and a 2 h relaxation step. Cyclic voltammetry (CV) and electrochemical impedance spectroscopy (EIS) measurements were carried out on a Solartron Analytical 1470E station (Ametek) with an amplitude of 10 mV at a frequency of 1000 kHz.

**Theoretical calculation**

The density functional theory (DFT) calculations were carried out with the VASP code [S1]. The Perdew-Burke-Ernzerhof (PBE) functional within generalized gradient approximation (GGA) was used to process the exchange-correlation [S2], while the projector-augmented-wave pseudopotential (PAW) was applied with a kinetic energy cut-off of 500 eV [S3], which was utilized to describe the expansion of the electronic eigenfunctions. The vacuum thickness was set to be 15 Å to minimize interlayer interactions. The Brillouin-zone integration was sampled by a Γ-centered 7 × 7 × 1 Monkhorst-Pack k-point. All atomic positions were fully relaxed until energy and force reached a tolerance of 1 × 10^-6^ eV and 0.01 eV/Å, respectively. The dispersion corrected DFT-D method was employed to consider the long-range interactions [S4]. Employing the climbing image nudged elastic band method (CI-NEB), we computed the minimum energy pathway of the diffuse reaction along with its corresponding activation barrier.

The Gibbs free energy change (ΔG) was calculated by computational hydrogen electrode (CHE) model as follows:

ΔG = ΔE + ΔZPE − TΔS (S1)

where ΔE is the reaction energy obtained by the total energy difference between the reactant and product molecules absorbed on the catalyst surface and ΔS is the change in entropy for each reaction, ΔZPE is the zero-point energy correction to the Gibbs free energy.

**S2 Supplementary Figures and Tables**


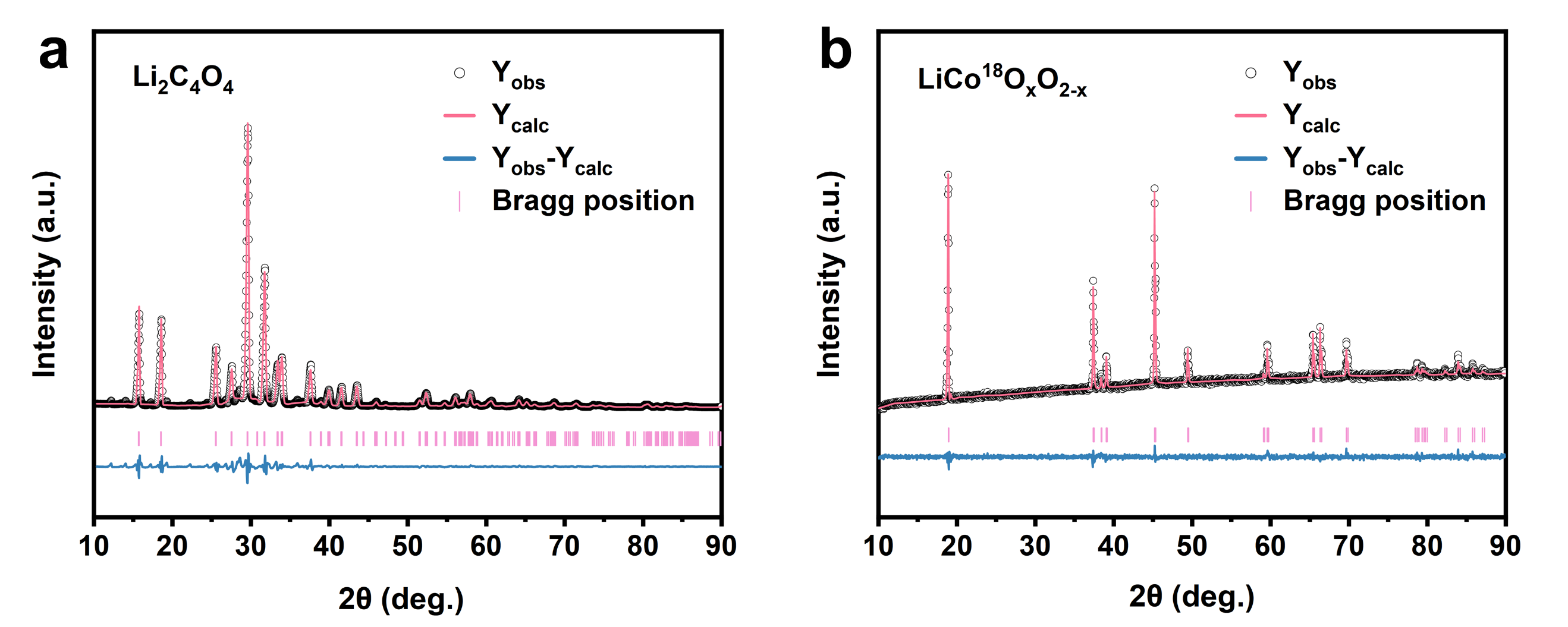


**Fig. S1** XRD and Rietveld refinement results of Li_2_C_4_O_4_ (a) and LiCo^18^O_x_O_2-x_ (b)


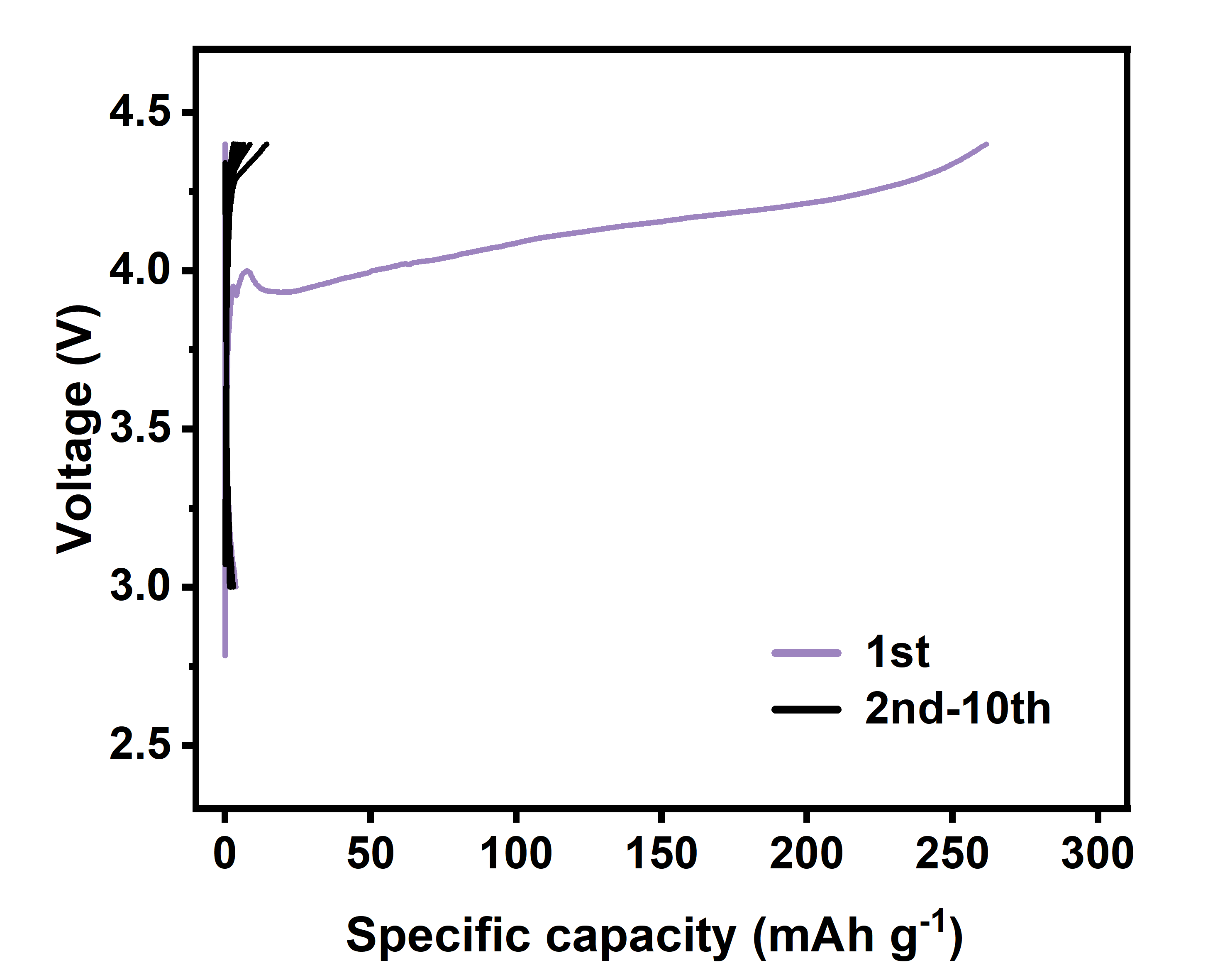


**Fig. S2** Charge-discharge curves of the first ten cycles of the DLS


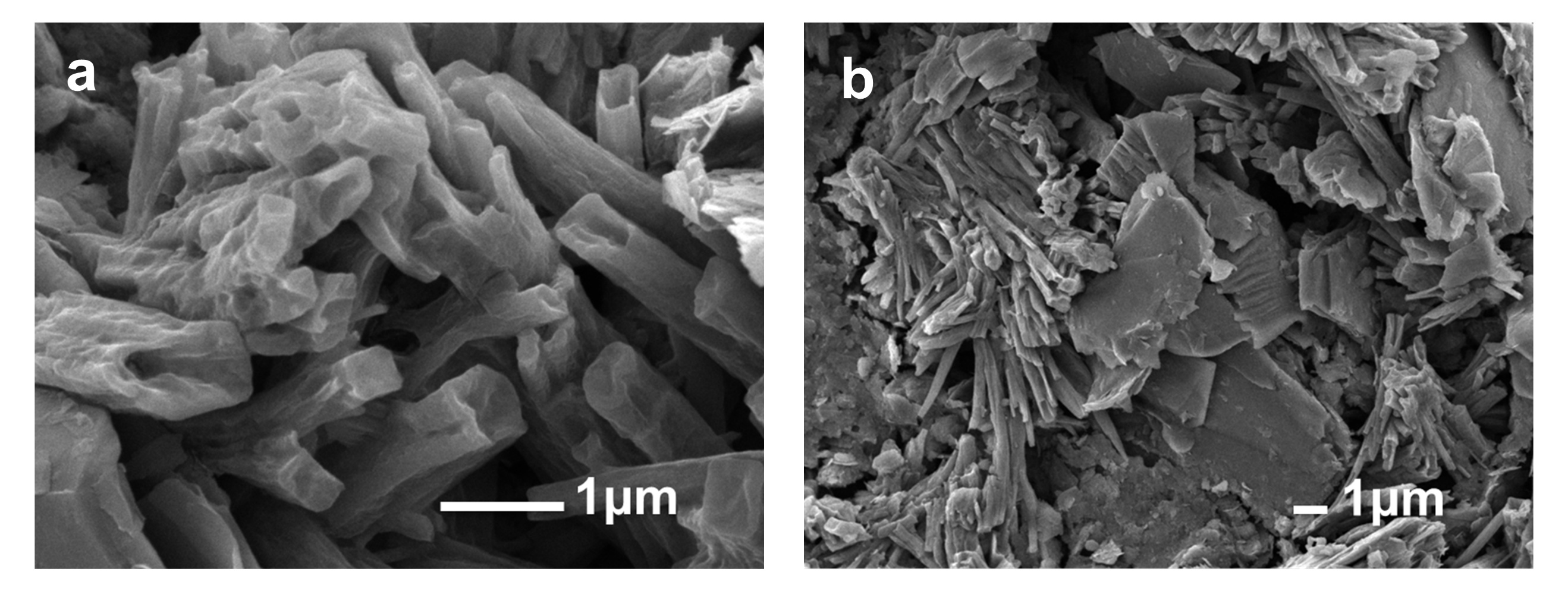


**Fig. S3** The SEM images of DLS (**a**) and GDLS (**b**)


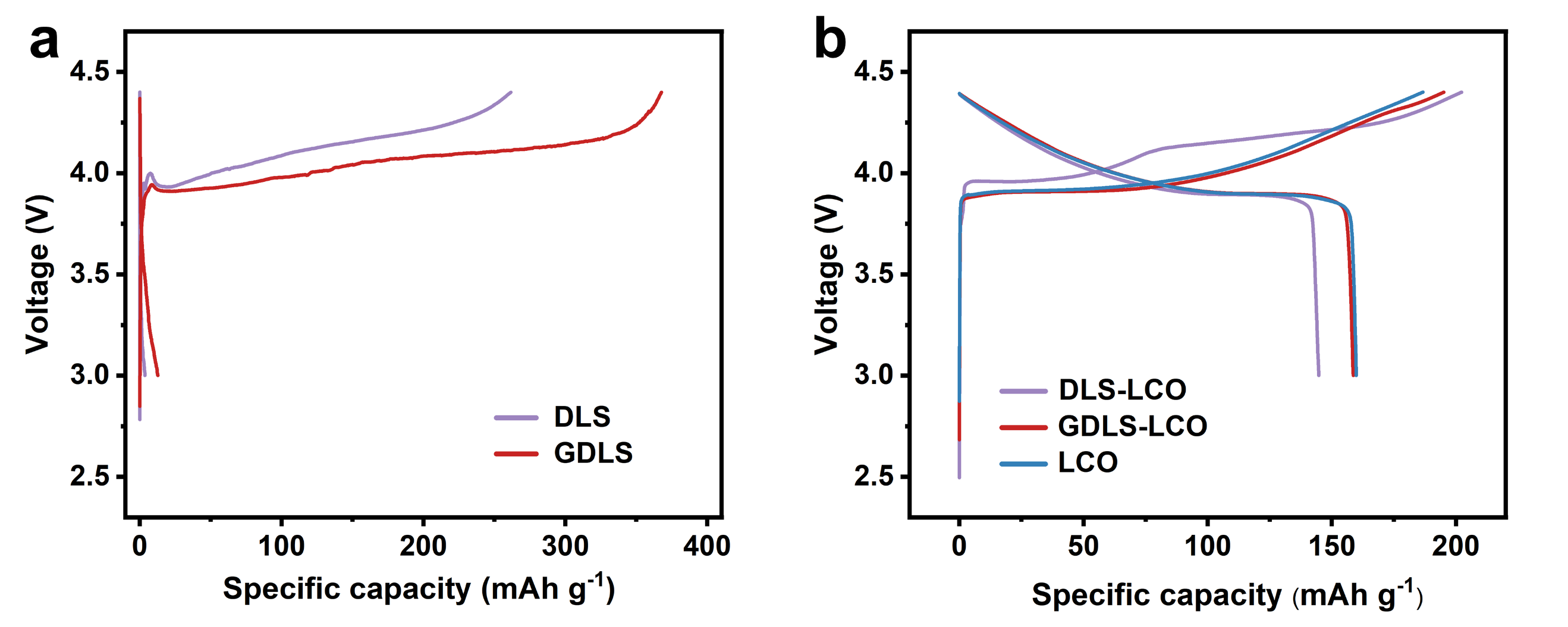


**Fig. S4** The first charge-discharge curves of DLS and GDLS (**a**), DLS-LCO, GDLS-LCO, and LCO (**b**)


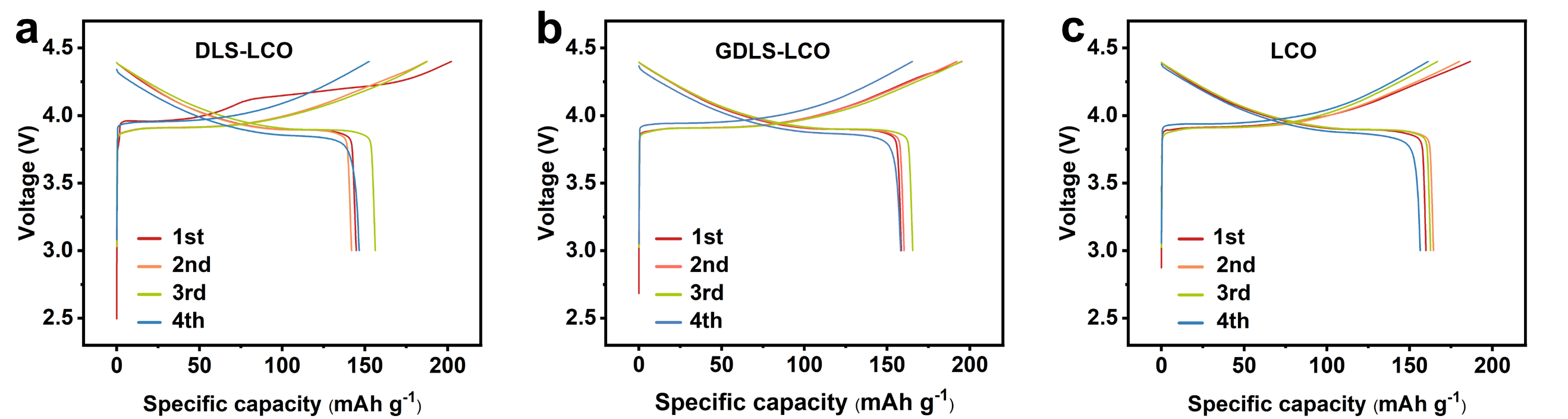


**Fig. S5** The charge-discharge curves of the first four cycles (0.1 C & 3 r + 1 C & 1 r) of DLS-LCO (**a**), GDLS-LCO (**b**), and LCO (**c**)


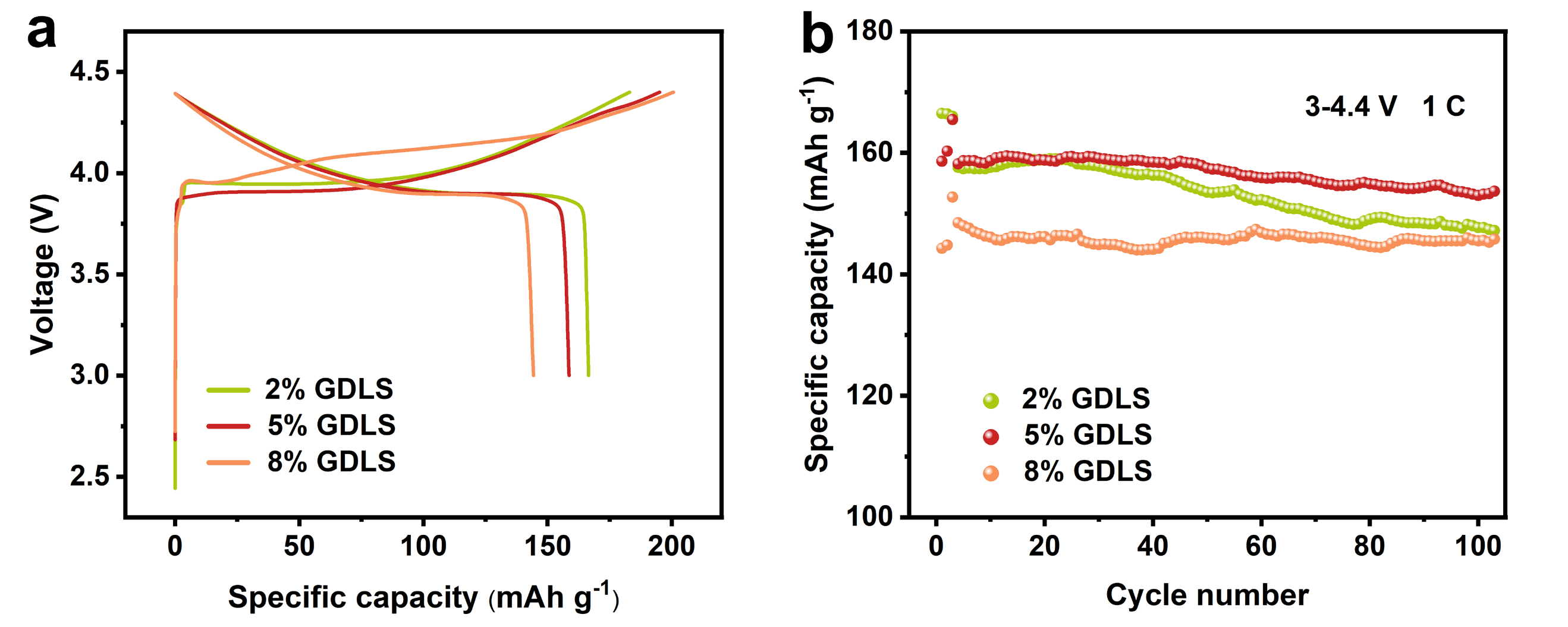


**Fig. S6** The first charge-discharge curves of LCO of 2% GDLS, 5% GDLS, and 8% GDLS were added separately (**a**), and their cyclic performance in the range of 3-4.4 V (**b**)


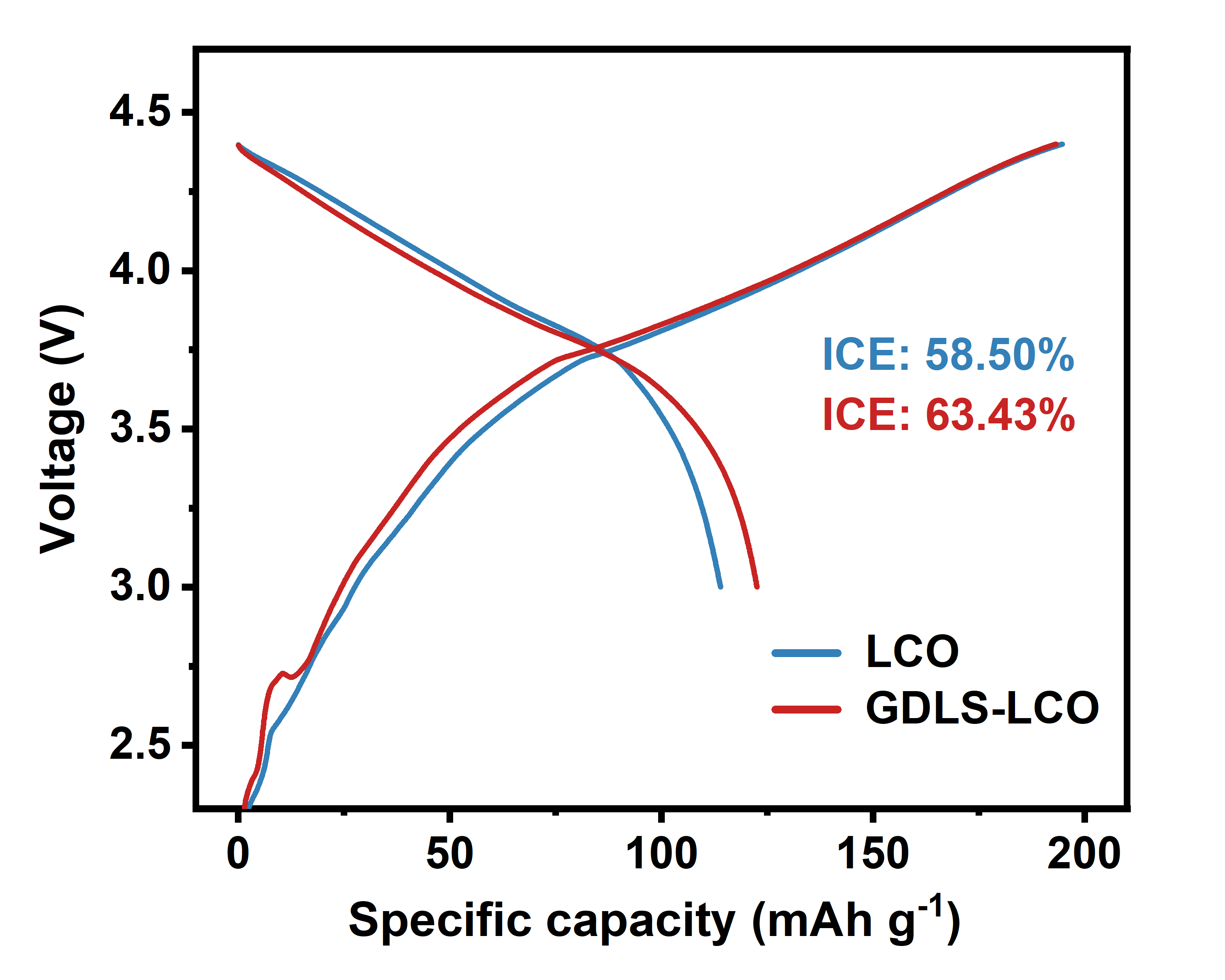


**Fig. S7** First-cycle charge-discharge curves of LCO||graphite and GDLS-LCO||graphite full-cells without offline prelithiation


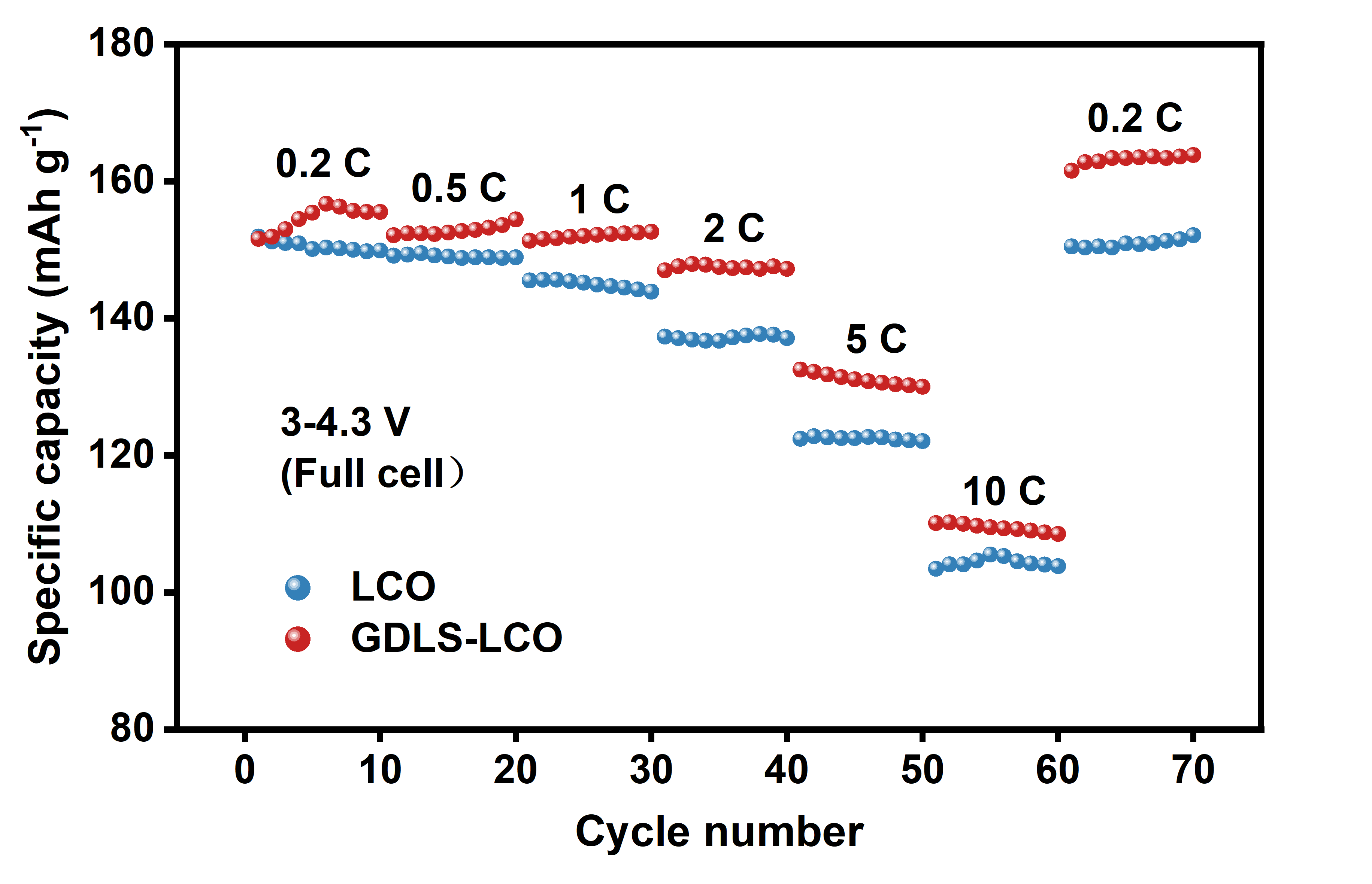


**Fig. S8** Rate performance of the LCO || graphite and GDLS-LCO || graphite full cells within 3-4.3 V


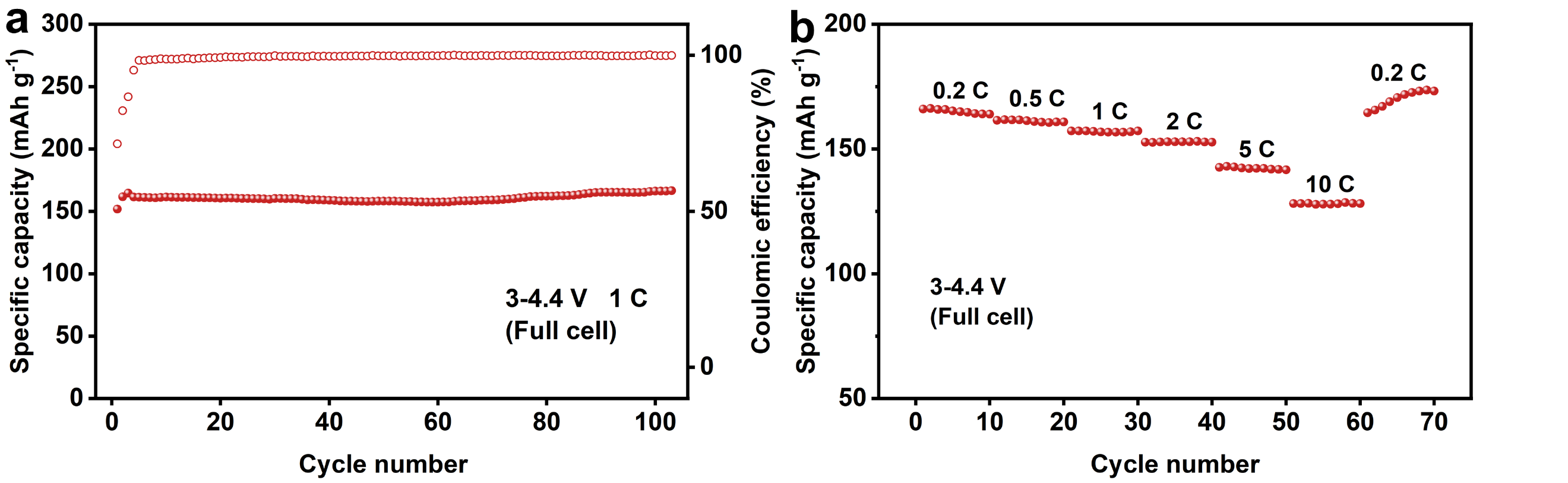


**Fig. S9** Cyclic performance (**a**) and rate performance (**b**) of the GDLS-LCO || graphite full cell within 3-4.4 V


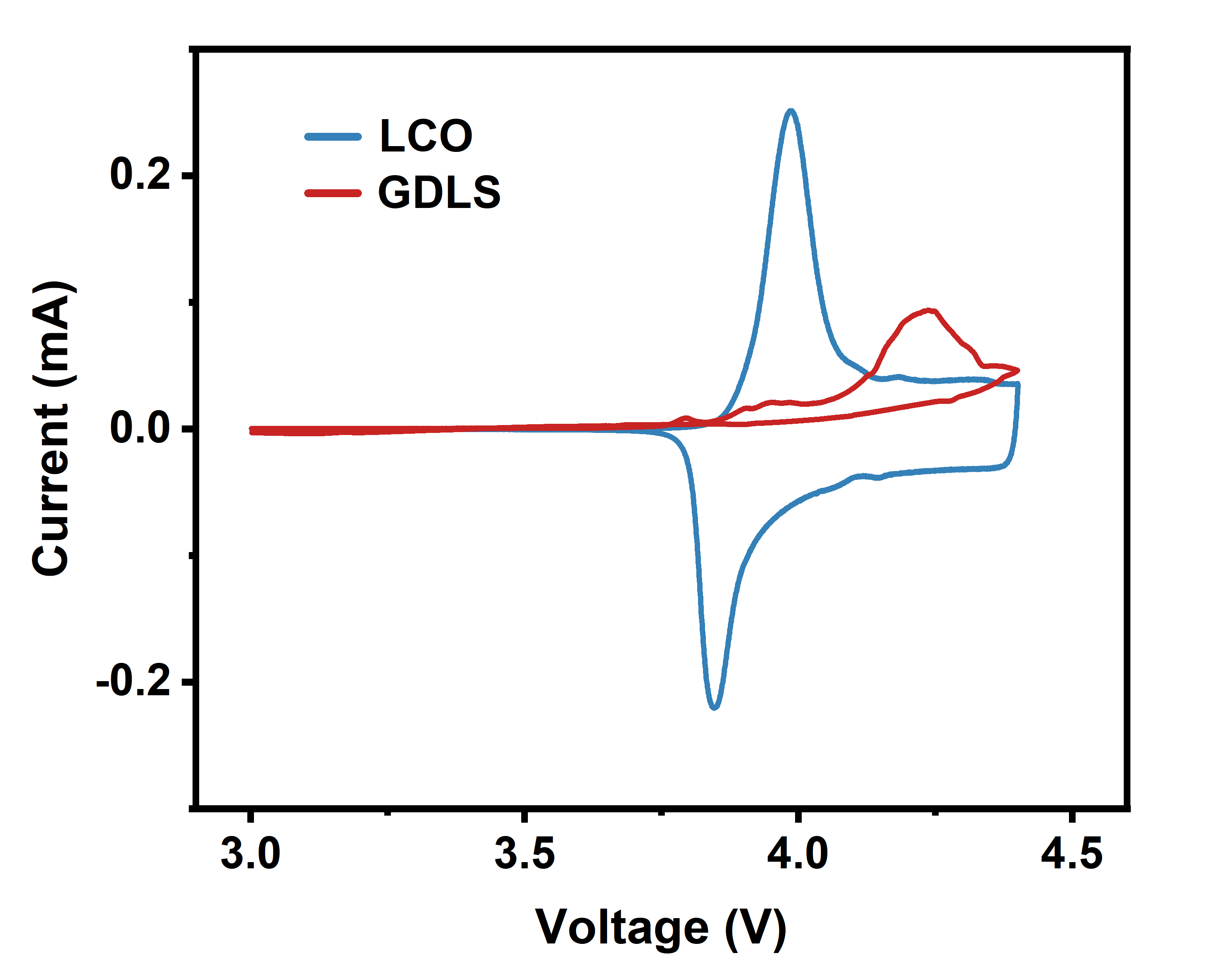


**Fig. S10** CV plots of LCO and GDLS at a scan rate of 0.03 mV s^-1^ within the voltage window of 3-4.4 V


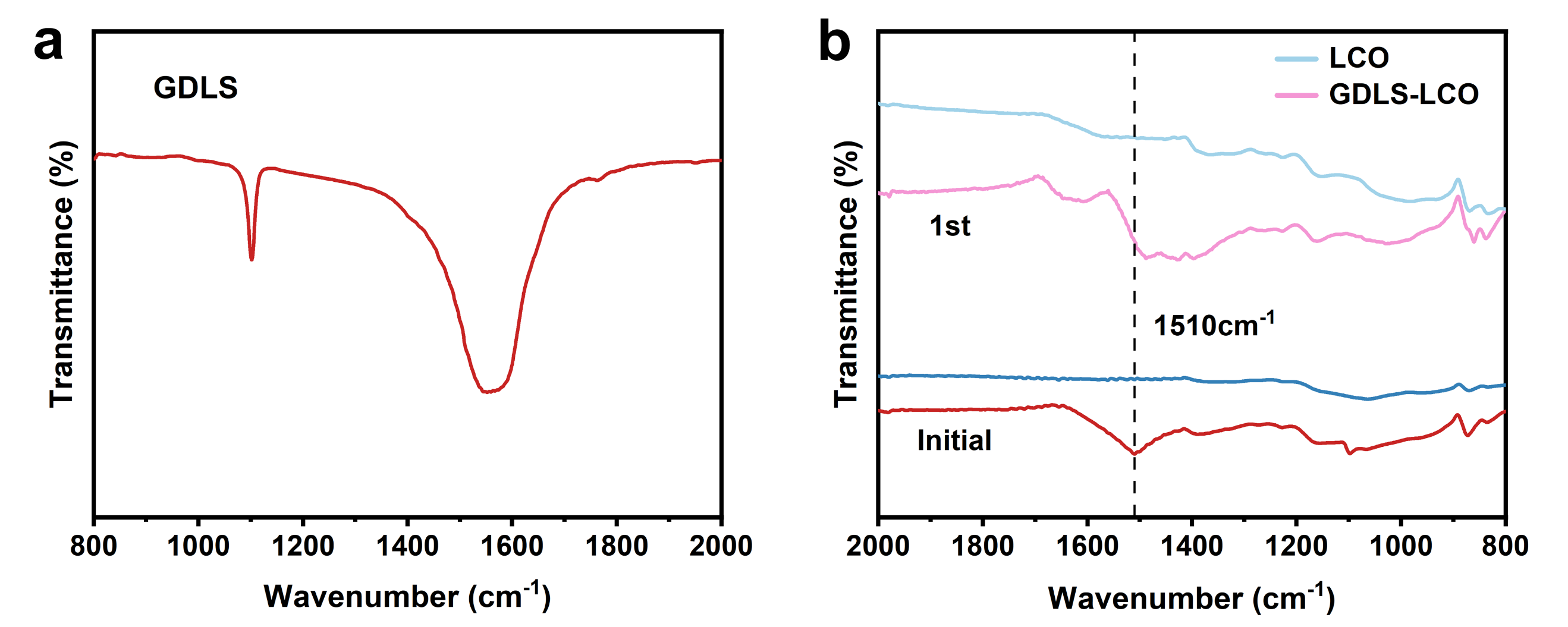


**Fig. S11** FTIR spectra of GDLS (**a**), LCO and GDLS-LCO electrodes before and after the first cycle (**b**)


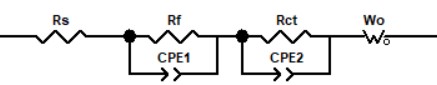


**Fig. S12** Equivalent circuit of EIS fitting


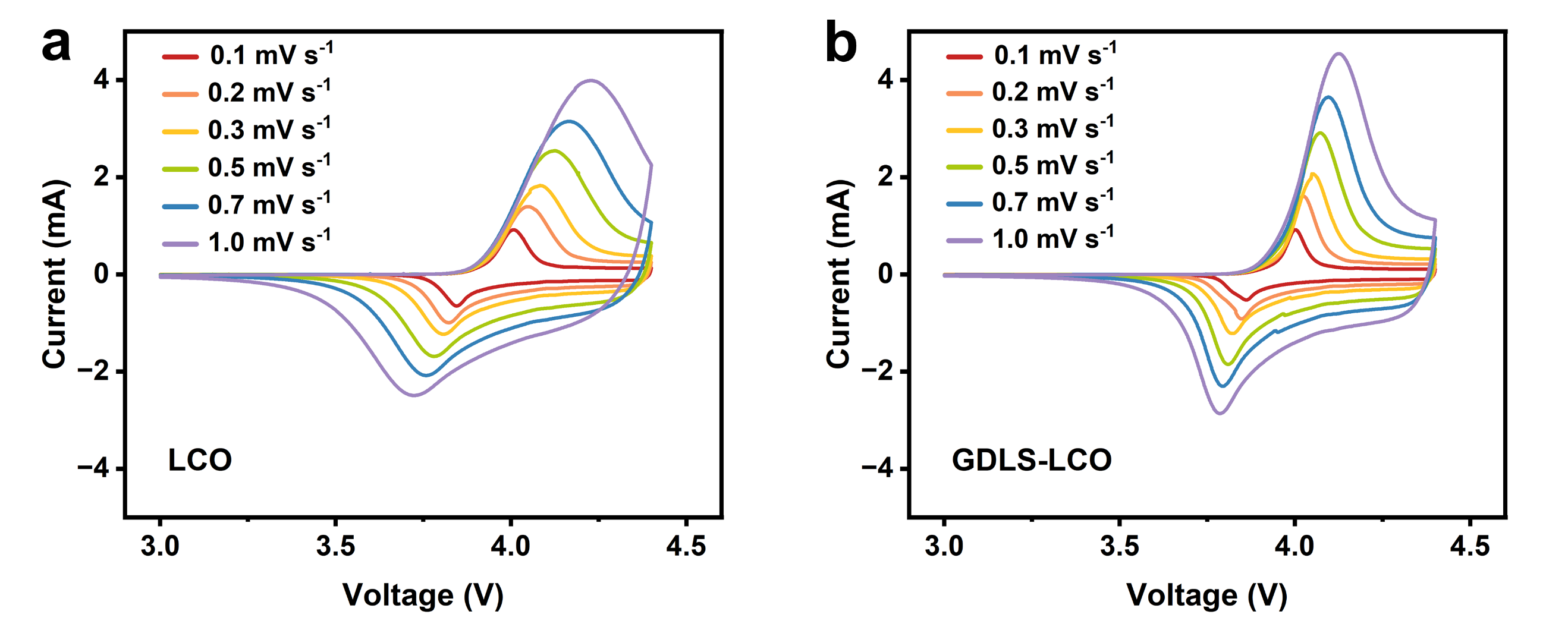


**Fig. S13** CV plots of LCO (**a**) and GDLS (**b**) at different scan rates within the voltage window of 3-4.4 V. To mitigate interference from GDLS presence, cells after 10-cycle aging at 1 C were selectively characterized


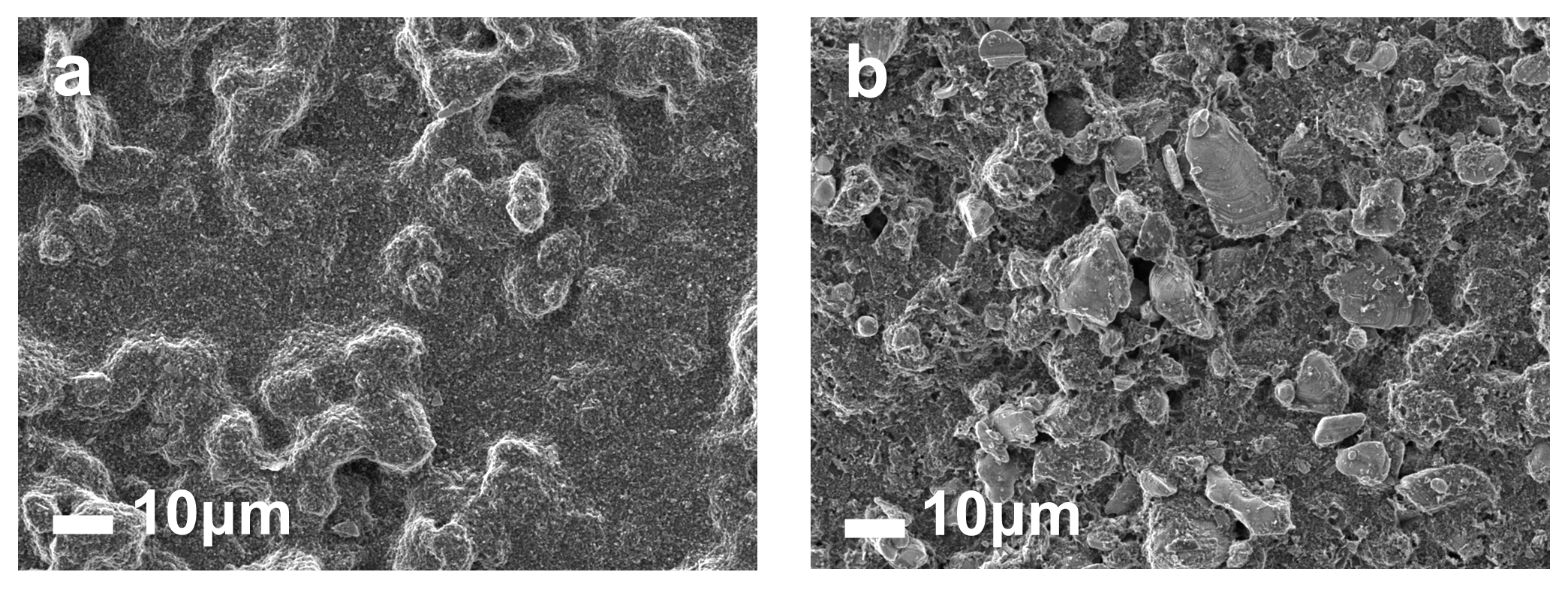


**Fig. S14** The SEM images of pristine cathodes of LCO (**a**) and GDLS-LCO (**b**)


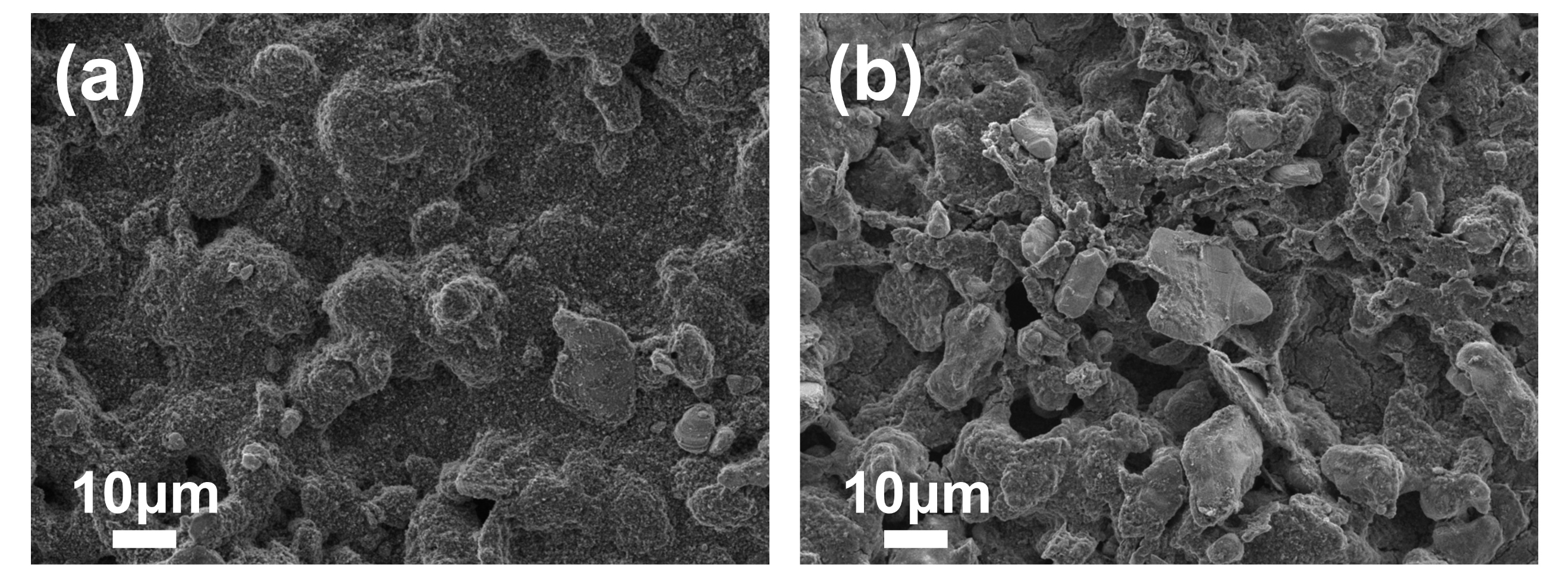


**Fig. S15** SEM images of LCO (**a**) and GDLS-LCO (**b**) cathodes after 600 cycles


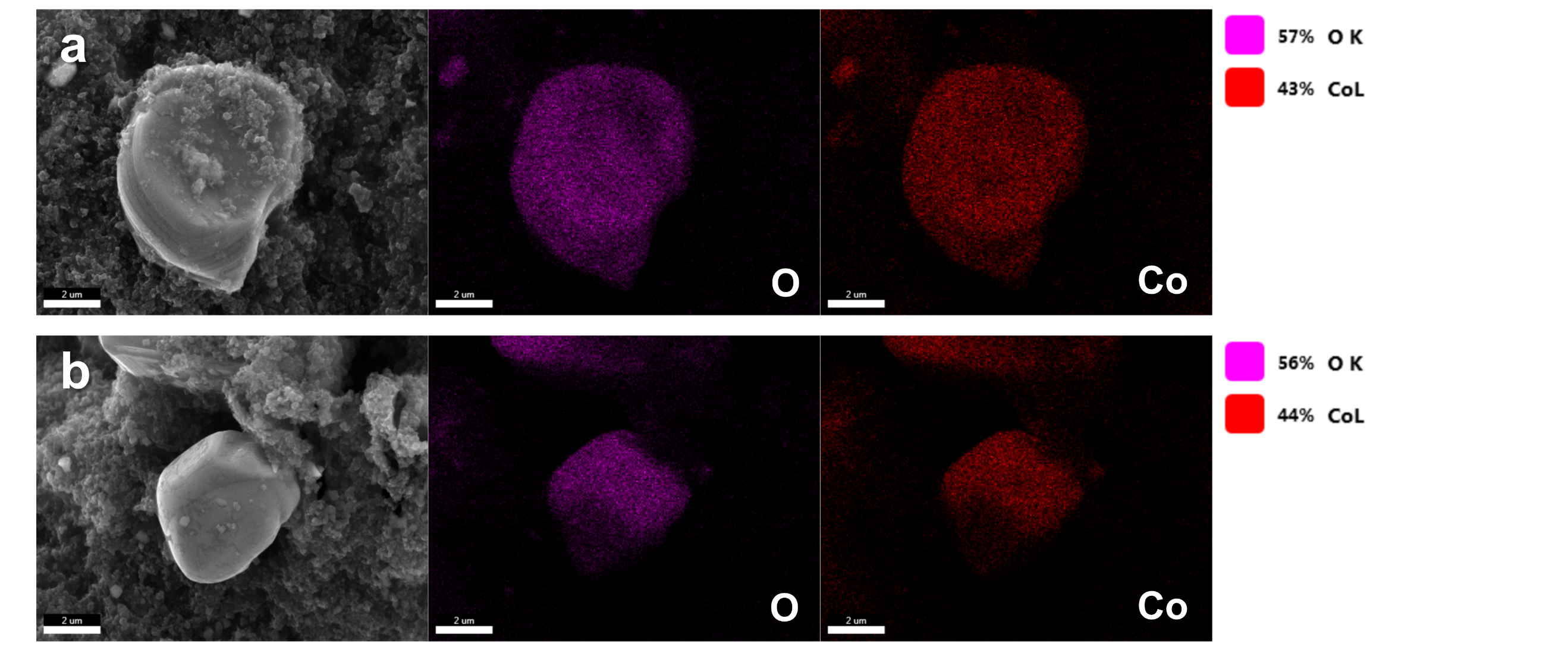


**Fig. S16** EDX mapping images of LiCoO_2_ particles in LCO (**a**) and GDLS-LCO (**b**) after 3 cycles at 0.1 C


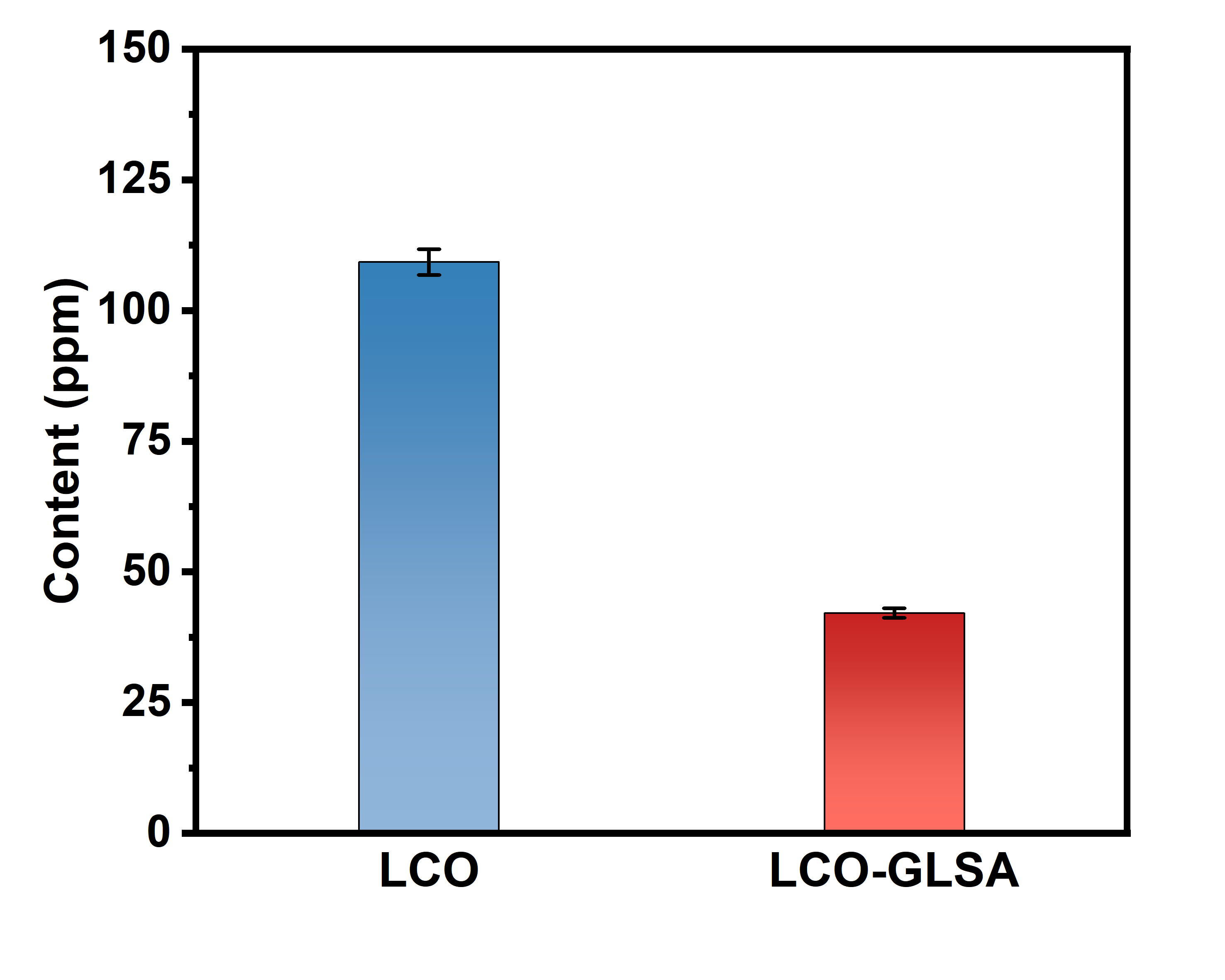


**Fig. S17** Co ion dissolution content in electrolyte measured by ICP-MS after 300 cycles


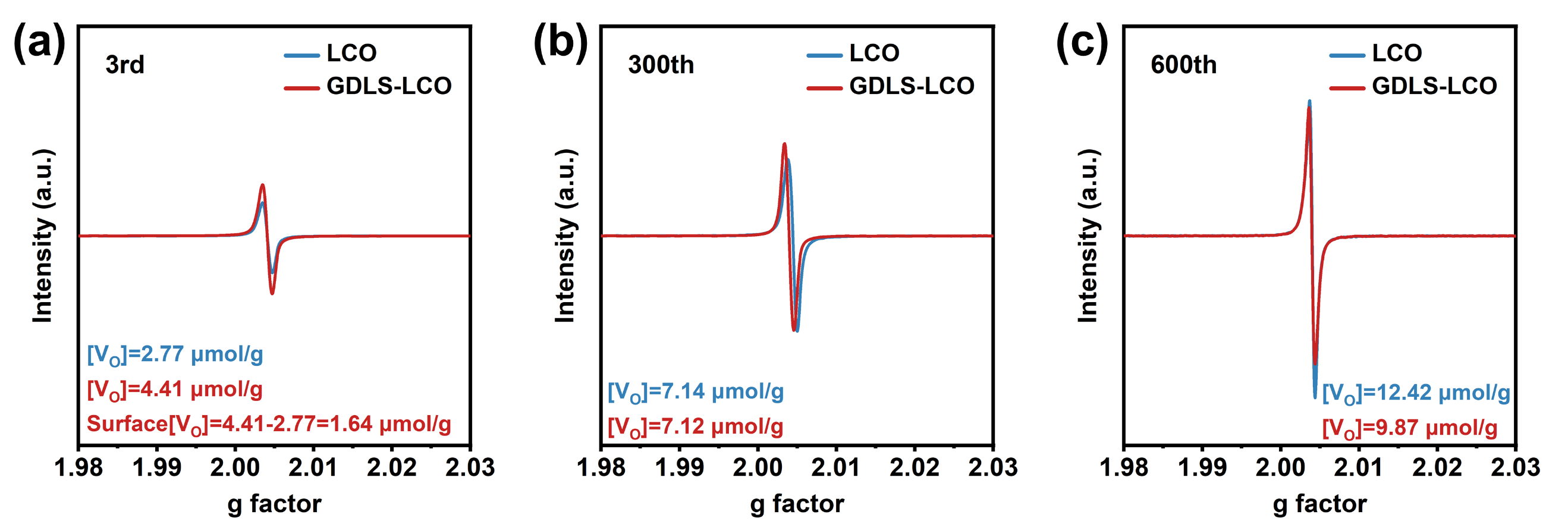


**Fig. S18** EPR spectra and quantitative analysis results of cathodes (without aluminum current collectors) after 3 cycles (**a**), 300 cycles (**b**), and 600 cycles (**c**)


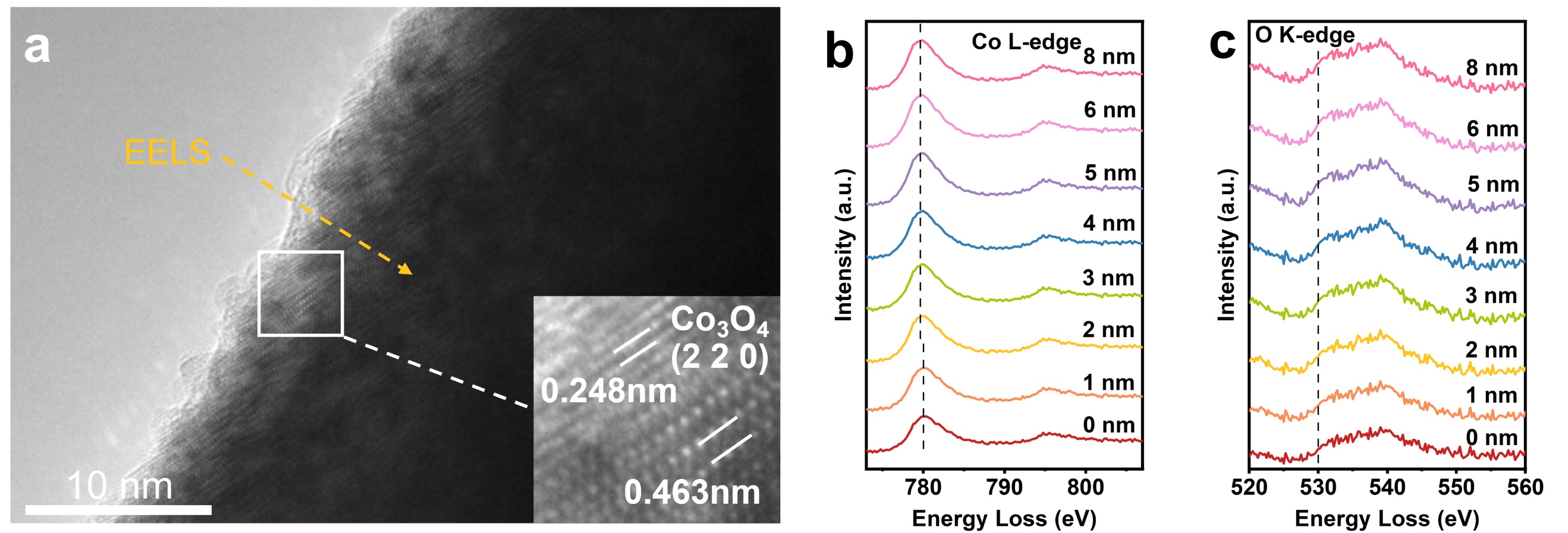


**Fig. S19** HRTEM images and corresponding near-surface EELS line scans (Co L-edge and O K-edge) of LiCoO₂ particles in GDLS-LCO after 600 cycles (**a-c**)


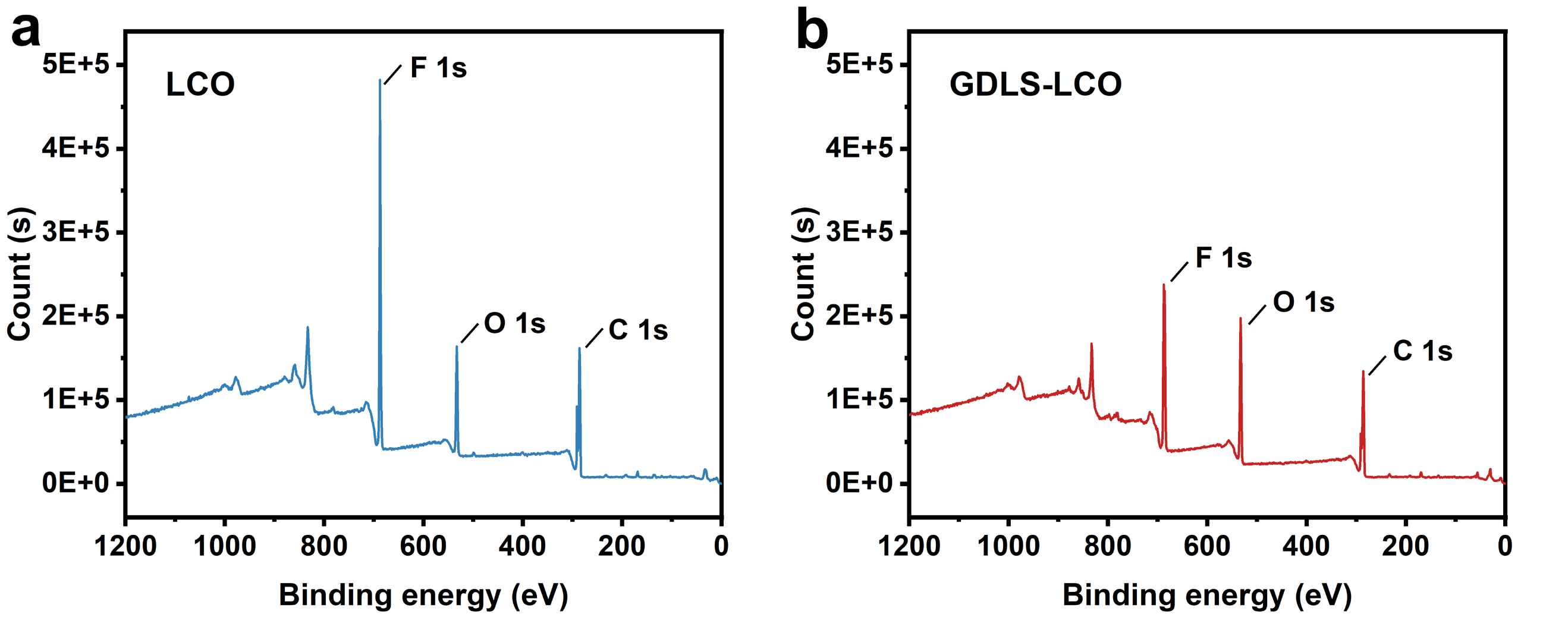


**Fig. S20** XPS survey spectra of cathode surfaces for LCO (**a**) and GDLS-LCO (**b**) after 300 cycles


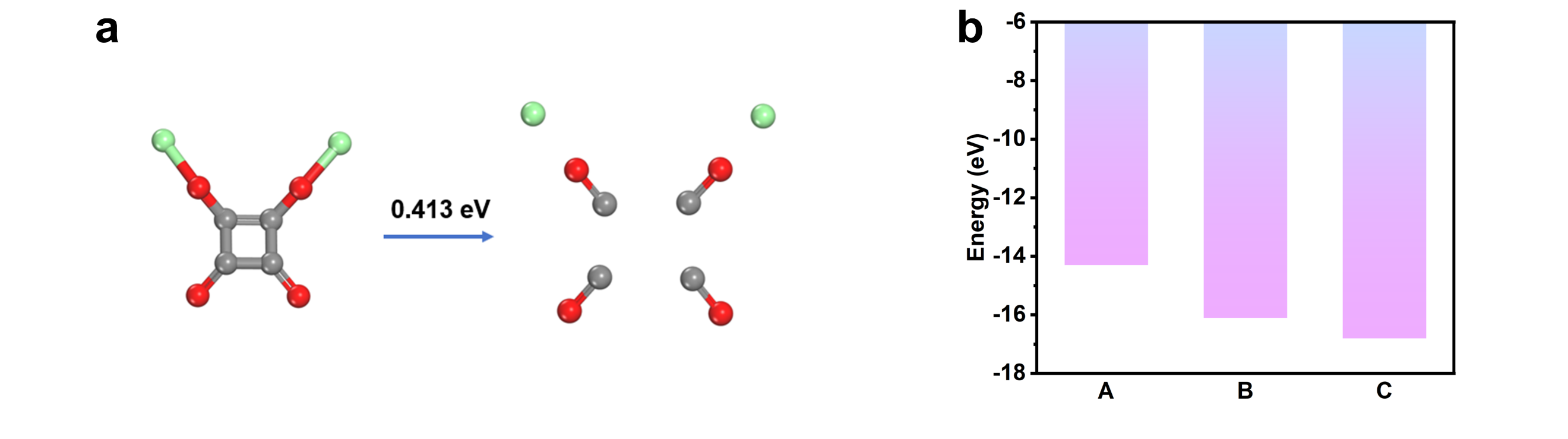


**Fig. S21** (**a**) Initial decomposition energy of DLS. (**b**)The energy differences among the three structures, $\dot{\cdot C}=O$ (A)，:C=O (B)，CO (C)


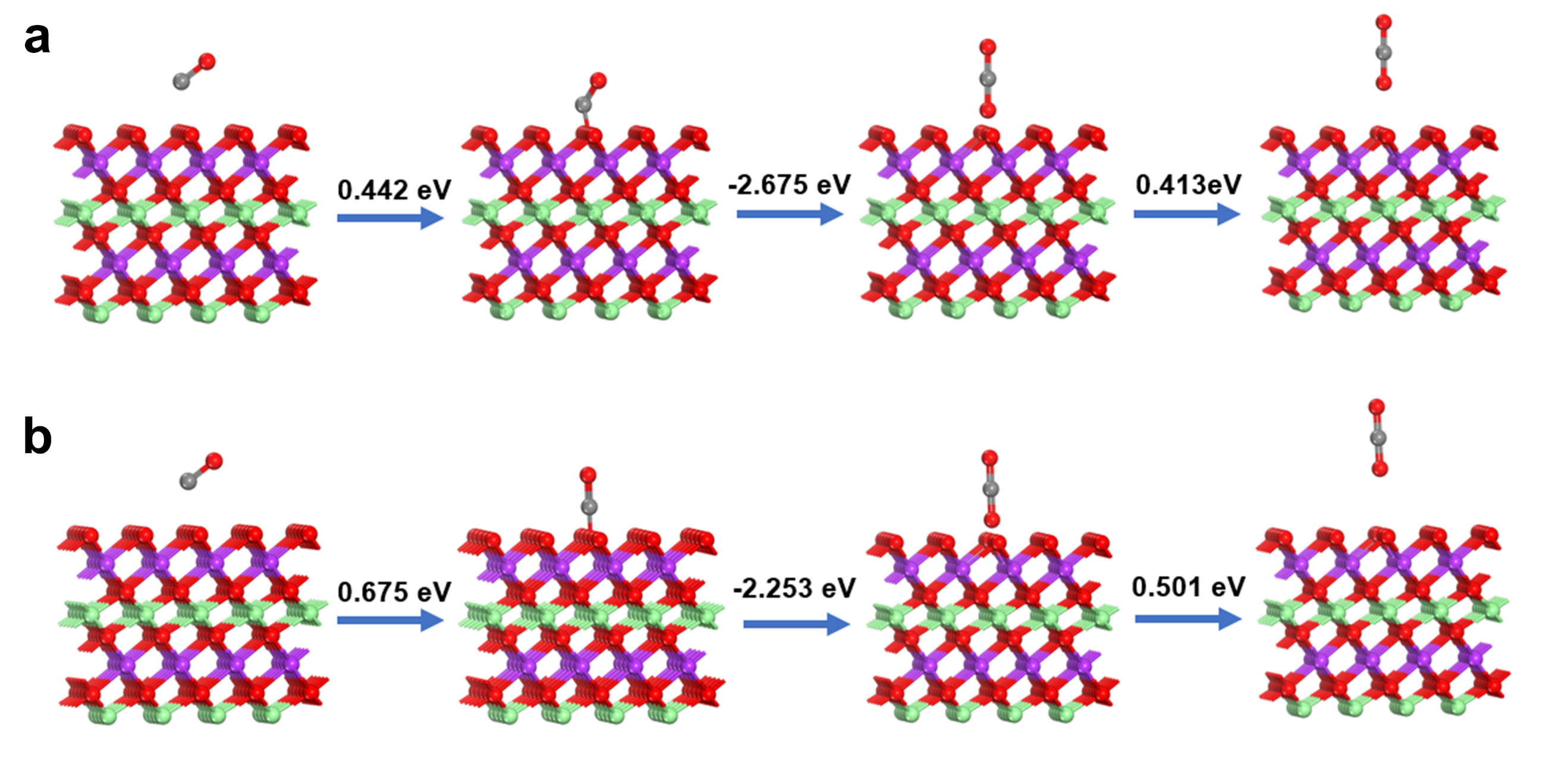


**Fig. S22** Reaction pathways and corresponding energy barriers for OV formation on the LCO surface mediated by :C=O (**a**) and CO (**b**). The process involves: adsorption of :C=O / CO, surface restructuring, and CO_2_ desorption


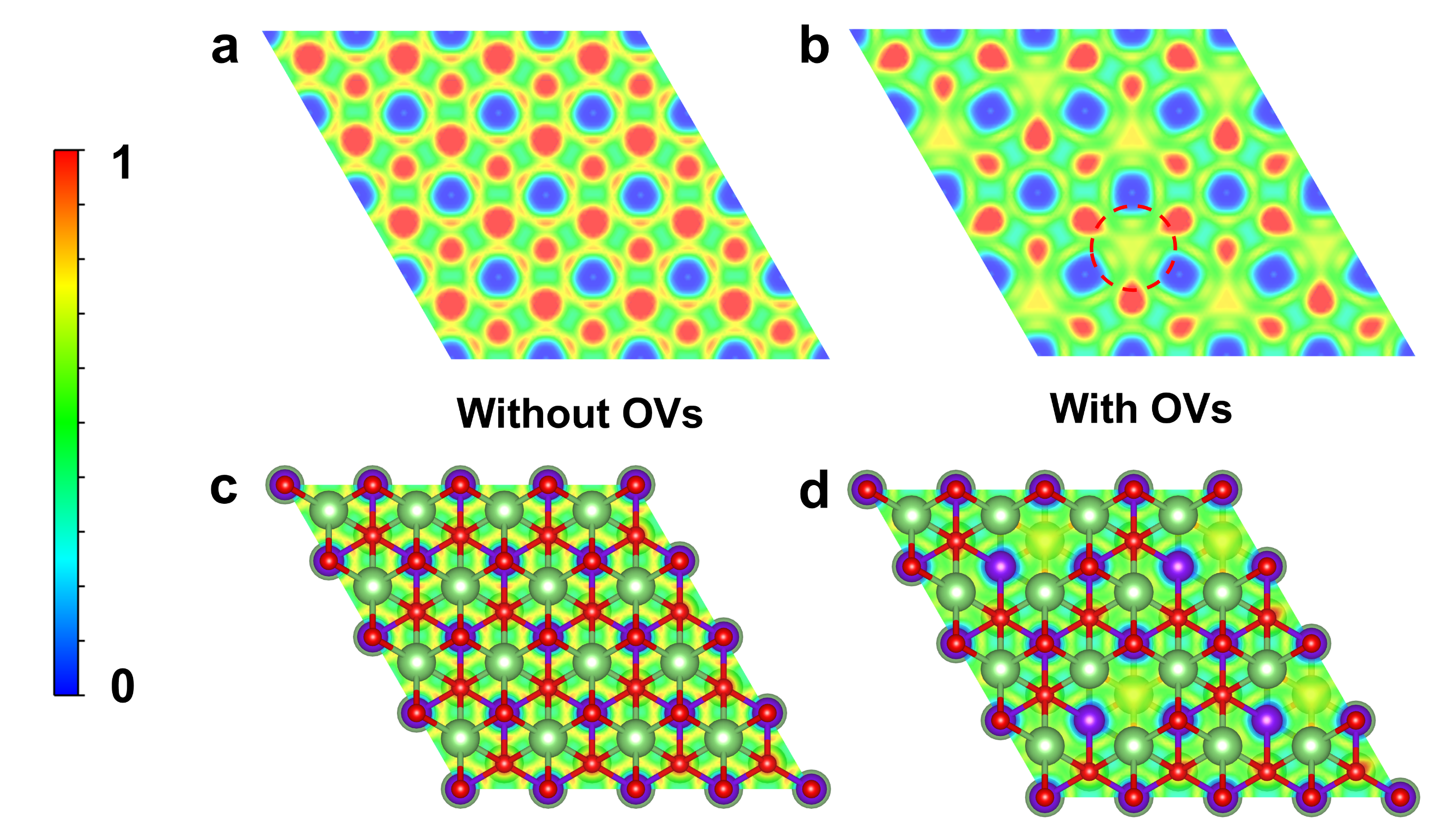


**Fig. S23** ELF plots for LCO without (**a, c**) and with (**b, d**) oxygen vacancies


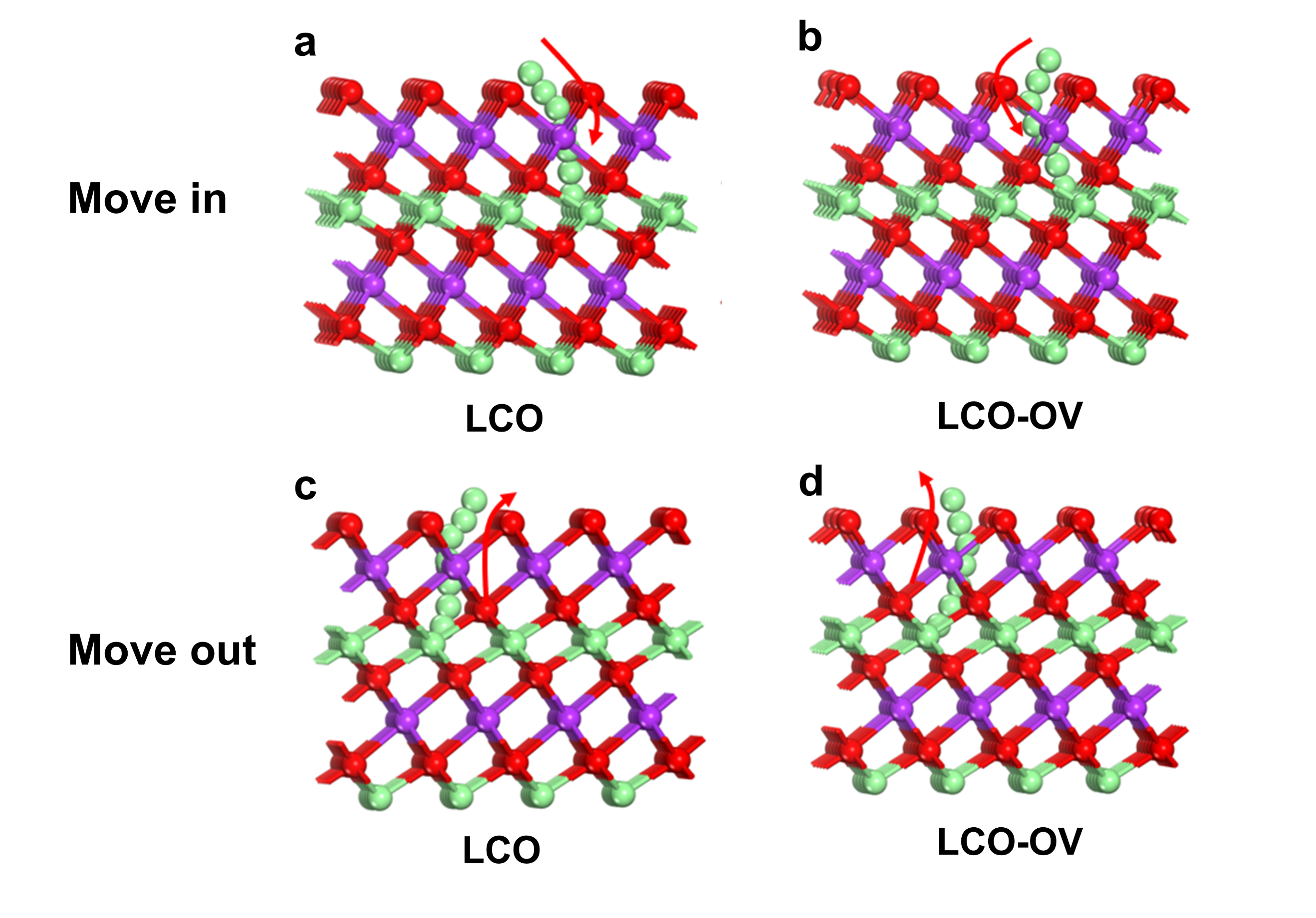


**Fig. S24** Li^+^ migration pathways into the LCO surface without (**a**) and with (**b**) OVs. Li^+^ migration pathways out of the LCO surface without (**c**) and with (**d**) OVs


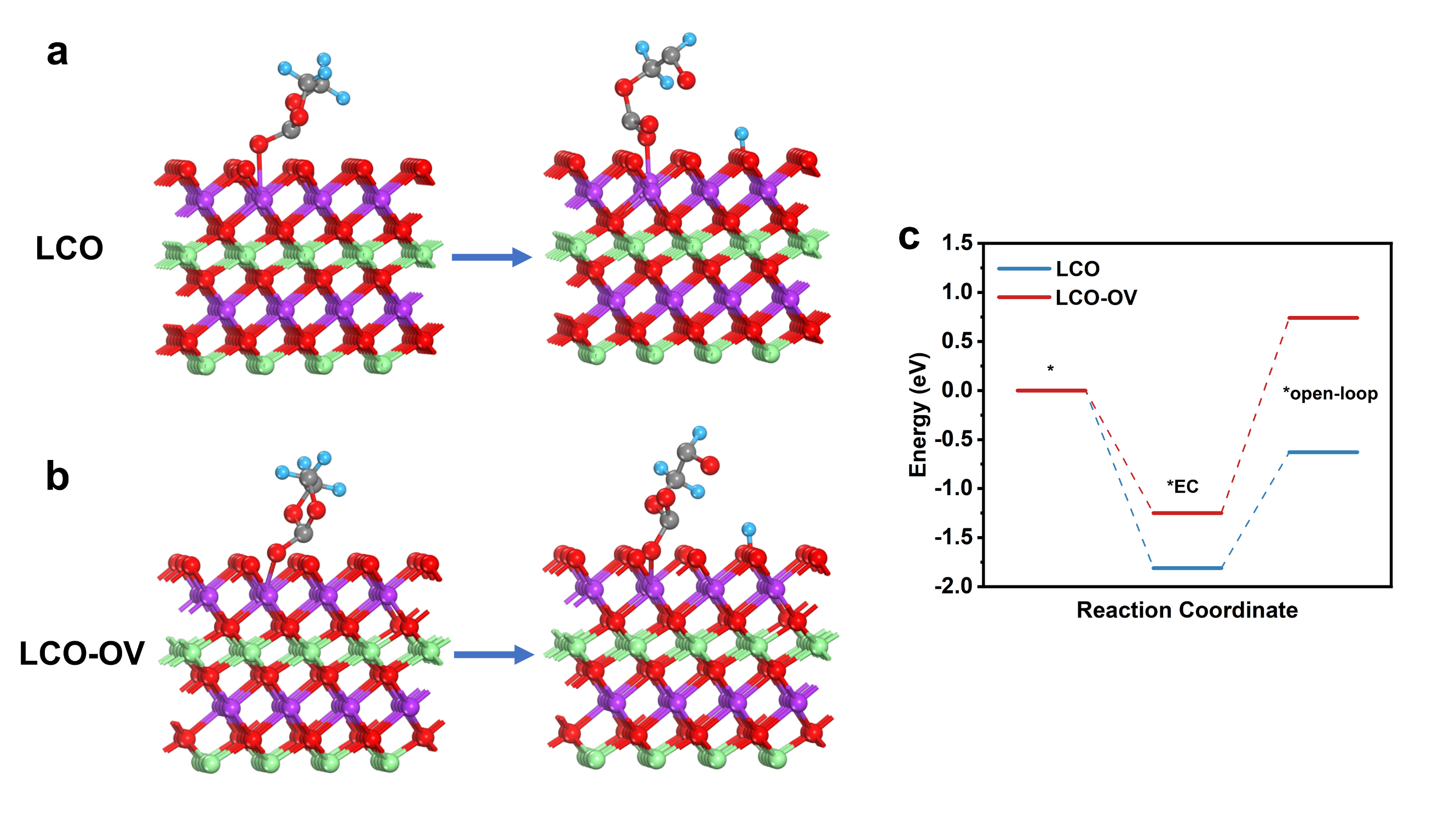


**Fig. S25** Schematic illustration of the ring-opening reaction of surface-adsorbed EC on LCO without (**a**) and with (**b**) OVs. Energy profiles for EC adsorption on LCO and LCO-OV surfaces and the subsequent ring-opening reaction (**c**)


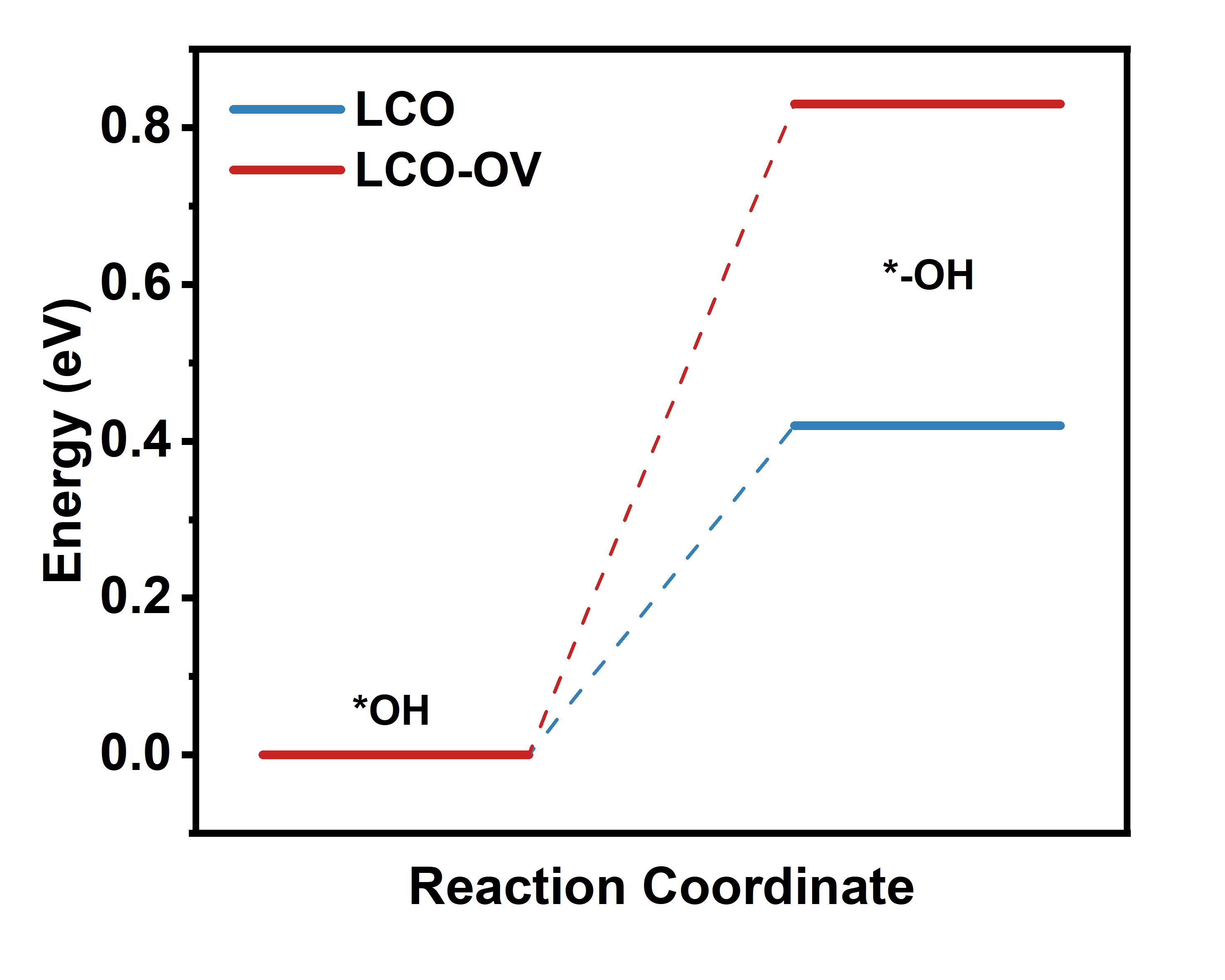


**Fig. S26** Desorption energy barriers of surface hydroxyl groups on LCO with and without oxygen vacancies


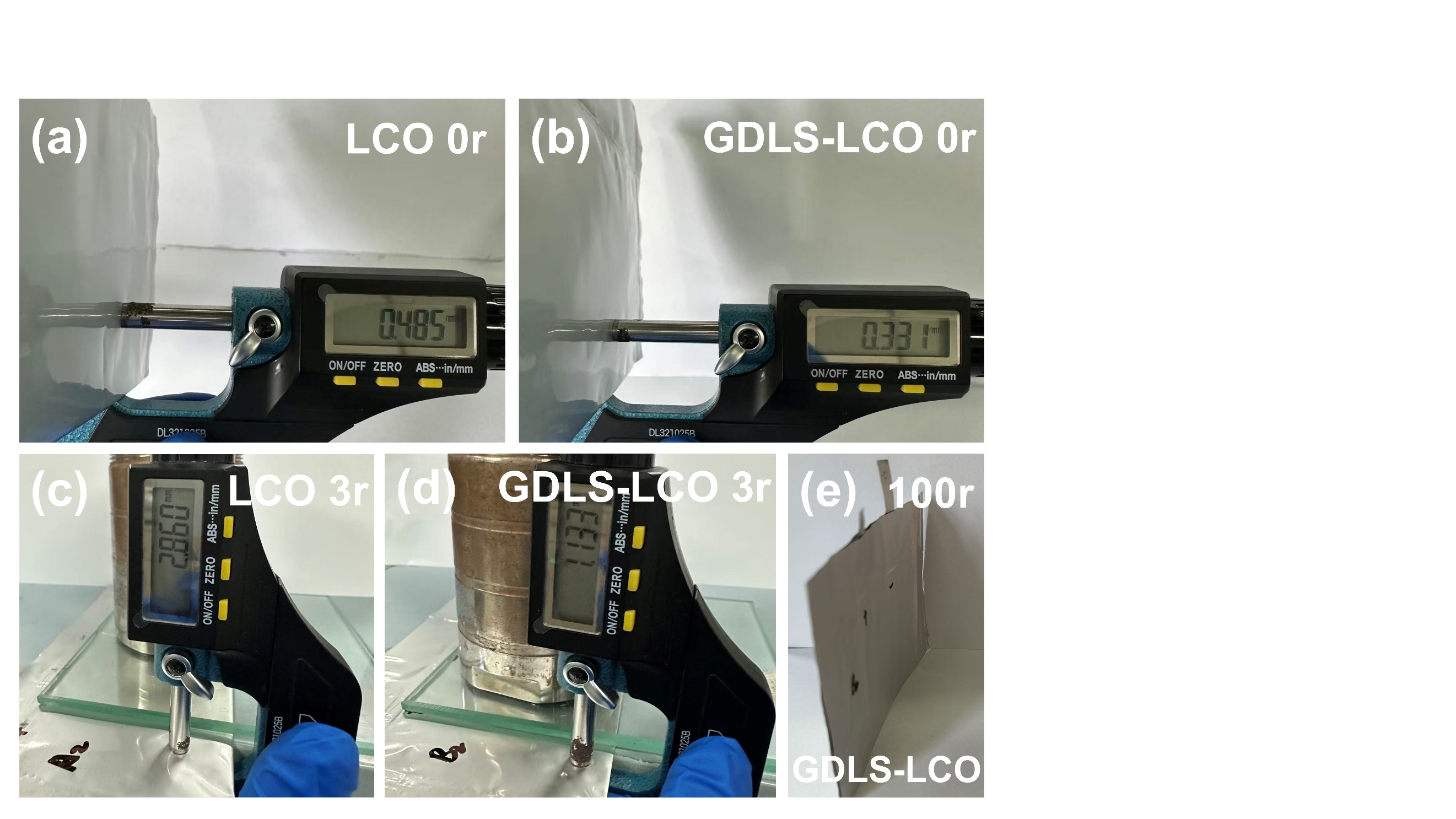


**Fig. S27** Thickness comparison of LCO‖graphite and GDLS-LCO‖graphite pouch cells before (**a, b**) and after (**c, d**) activation, and the photograph of GDLS-LCO‖graphite pouch cell after 100 cycles (**e**)


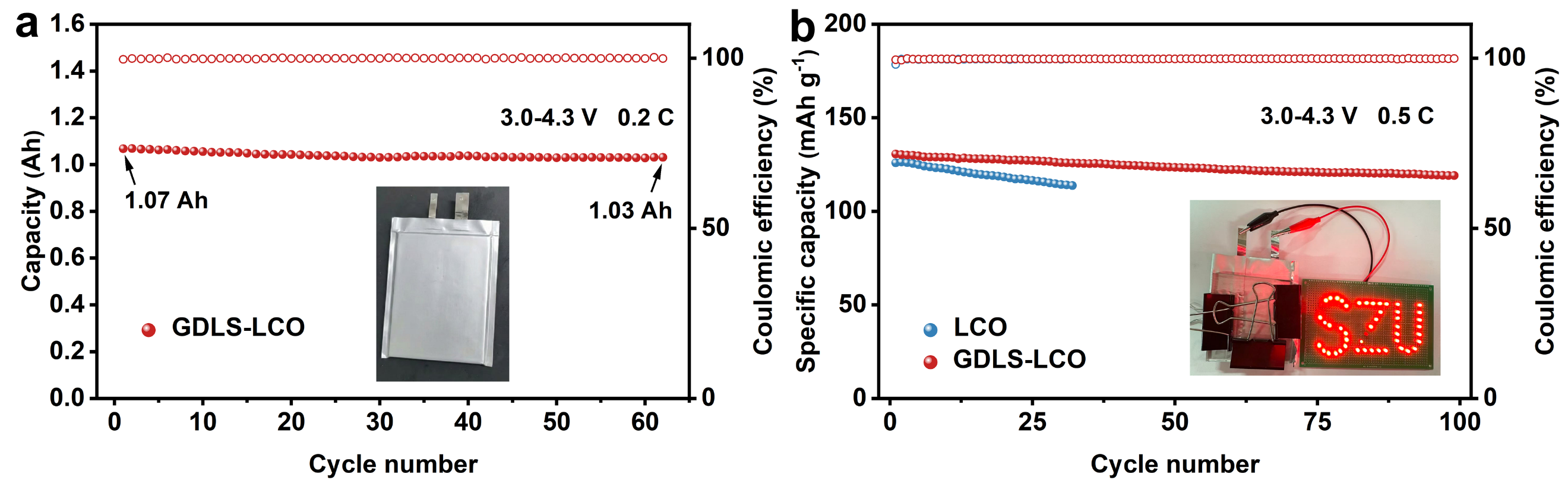


**Fig. S28** Cycling performance of high-capacity GDLS-LCO‖Li pouch cells (**a**); Cycling performance of LCO‖graphite and GDLS-LCO‖graphite pouch cells (**b**)


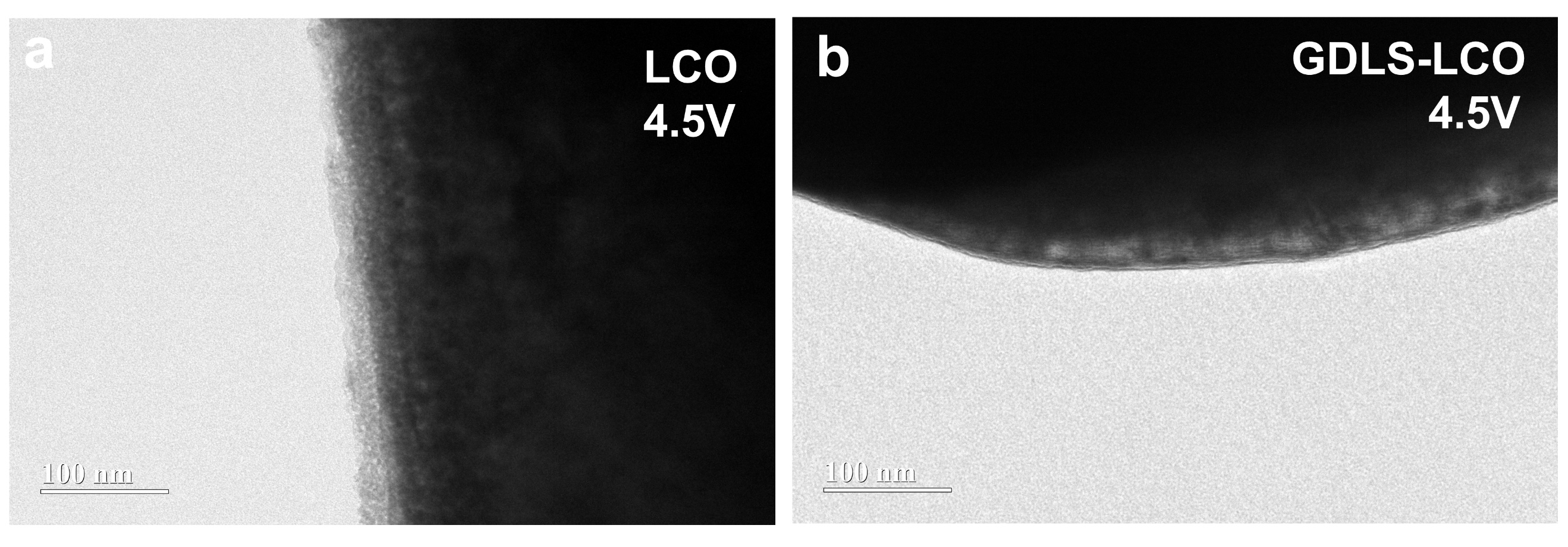


**Fig. S29** TEM images of LCO (**a**) and GDLS-LCO (**b**) after 300 cycles at 3.0–4.5 V


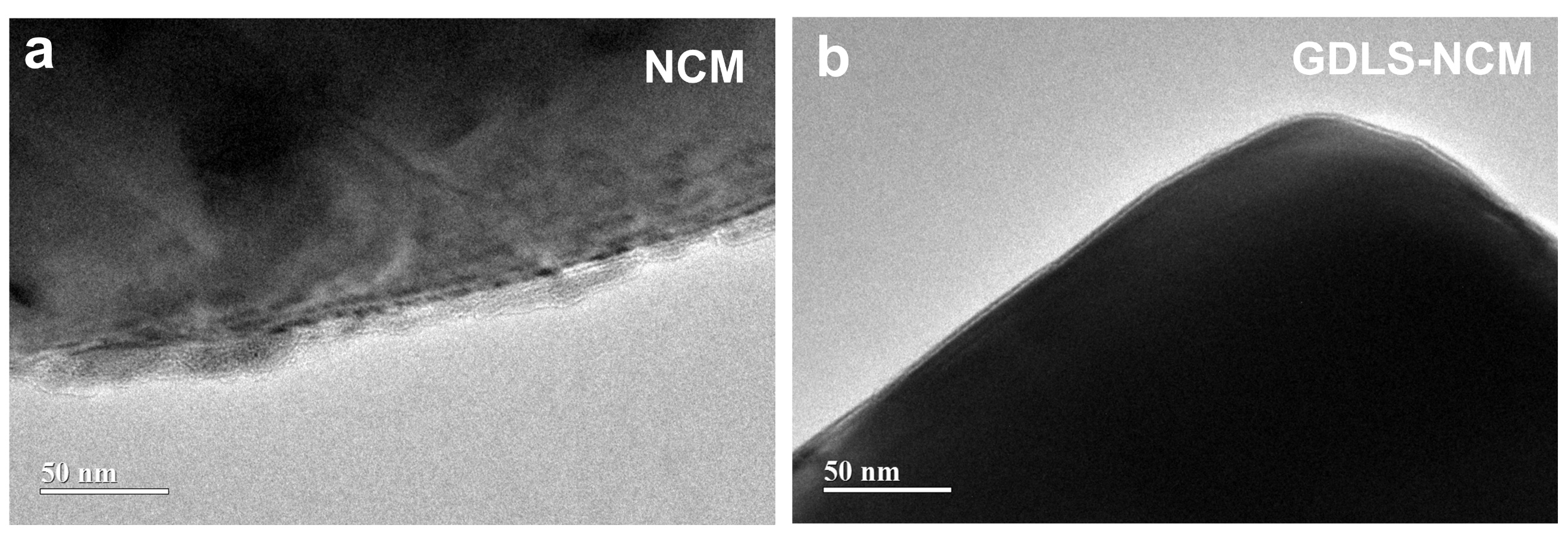


**Fig. S30** TEM images of NCM (a) and GDLS-NCM (b) after 300 cycles at 3.0–4.5 V

**Table S1** Lattice parameters of the Li_2_C_4_O_4_ and LiCo^18^O_x_O_2-x_ are obtained through Fullprof

| Lattice parameter | a | b | c |
| --- | --- | --- | --- |
| Li_2_C_4_O_4_ | 7.1163 | 9.5735 | 3.3030 |
| LiCo^18^O_x_O_2-x_ | 2.8166 | 2.8166 | 14.0450 |

**Table S2** The summarization of peak position and corresponding compounds ratio in C 1s

| Peaks | Positions (eV) | Ratio (%)  -LCO | Ratio (%)  -GDLS-LCO |
| --- | --- | --- | --- |
| Li_2_CO_3_ | 290.99 | 23.01 | 16.72 |
| O=C-O | 288.86 | 4.70 | 7.36 |
| C=O | 287.86 | 2.79 | 7.74 |
| C-O | 286.45 | 50.53 | 57.24 |
| C-C | 285.00 | 18.97 | 10.93 |

**Table S3** The summarization of peak position and corresponding compounds ratio in O 1s

| Peaks | Positions (eV) | Ratio (%)  -LCO | Ratio (%)  -GDLS-LCO |
| --- | --- | --- | --- |
| Li_x_PF_y_O_z_ | 534.79 | 4.97 | 5.13 |
| C-O | 533.58 | 47.64 | 44.68 |
| C=O | 532.53 | 30.67 | 35.30 |
| P-O | 531.55 | 16.73 | 14.89 |

**Table S4** The summarization of peak position and corresponding compounds ratio in F 1s

| Peaks | Positions (eV) | Ratio (%)  -LCO | Ratio (%)  -GDLS-LCO |
| --- | --- | --- | --- |
| C-F/LiPF_6_ | 688.16 | 58.45 | 40.76 |
| Li_x_PF_y_O_z_ /Li_x_PF_y_ | 687.43 | 30.06 | 11.51 |
| LiF | 685.67 | 11.48 | 47.73 |

**Supplementary References**

1. G. Kresse, J. Furthmüller. Efficiency of ab-initio total energy calculations for metals and semiconductors using a plane-wave basis set. Comput. Mater. Sci. **6**(1), 15-50 (1996). <https://doi.org/10.1016/0927-0256(96)00008-0>
2. J. P. Perdew, K. Burke, M. Ernzerhof. Generalized gradient approximation made simple. Phys. Rev. Lett. **77**(18), 3865-3868 (1996). [https://doi.org/10.1103/PhysRevLett.77.3865](%20https:/doi.org/10.1103/PhysRevLett.77.3865)
3. P. E. Blöchl. Projector augmented-wave method. Phys. Rev. B. **50**(24), 17953-17979 (1994). <https://doi.org/10.1103/PhysRevB.50.17953>
4. S. Grimme. Semiempirical gga-type density functional constructed with a long-range dispersion correction. J. Comput. Chem. **27**(15), 1787-1799 (2006). <https://doi.org/10.1002/jcc.20495>
